# FGF receptors mediate cellular senescence in the cystic fibrosis airway epithelium

**DOI:** 10.1172/jci.insight.174888

**Published:** 2024-06-25

**Authors:** Molly Easter, Meghan June Hirsch, Elex Harris, Patrick Henry Howze, Emma Lea Matthews, Luke I. Jones, Seth Bollenbecker, Shia Vang, Daniel J. Tyrrell, Yan Y. Sanders, Susan E. Birket, Jarrod W. Barnes, Stefanie Krick

**Affiliations:** 1 Division of Pulmonary, Allergy and Critical Care Medicine, Department of Medicine;; 2 Gregory Fleming James Cystic Fibrosis Research Center, and; 3 Division of Molecular and Cellular Pathology, Department of Pathology, The University of Alabama at Birmingham (UAB), Birmingham, Alabama, USA.; 4 Eastern Virginia Medical School, Norfolk, Virginia, USA.

**Keywords:** Cell biology, Pulmonology, Cellular senescence

## Abstract

The number of adults living with cystic fibrosis (CF) has already increased significantly because of drastic improvements in life expectancy attributable to advances in treatment, including the development of highly effective modulator therapy. Chronic airway inflammation in CF contributes to morbidity and mortality, and aging processes like inflammaging and cell senescence influence CF pathology. Our results show that single-cell RNA sequencing data, human primary bronchial epithelial cells from non-CF and CF donors, a CF bronchial epithelial cell line, and *Cftr*-knockout (*Cftr^–/–^*) rats all demonstrated increased cell senescence markers in the CF bronchial epithelium. This was associated with upregulation of fibroblast growth factor receptors (FGFRs) and mitogen-activated protein kinase (MAPK) p38. Inhibition of FGFRs, specifically FGFR4 and to some extent FGFR1, attenuated cell senescence and improved mucociliary clearance, which was associated with MAPK p38 signaling. Mucociliary dysfunction could also be improved using a combination of senolytics in a CF ex vivo model. In summary, FGFR/MAPK p38 signaling contributes to cell senescence in CF airways, which is associated with impaired mucociliary clearance. Therefore, attenuation of cell senescence in the CF airways might be a future therapeutic strategy improving mucociliary dysfunction and lung disease in an aging population with CF.

## Abstract 2

Cellular senescence in the cystic fibrosis bronchial epithelium is partially mediated by fibroblast growth factor receptors via p38 MAPK signaling and independent of modulator therapy.

## Graphical abstract

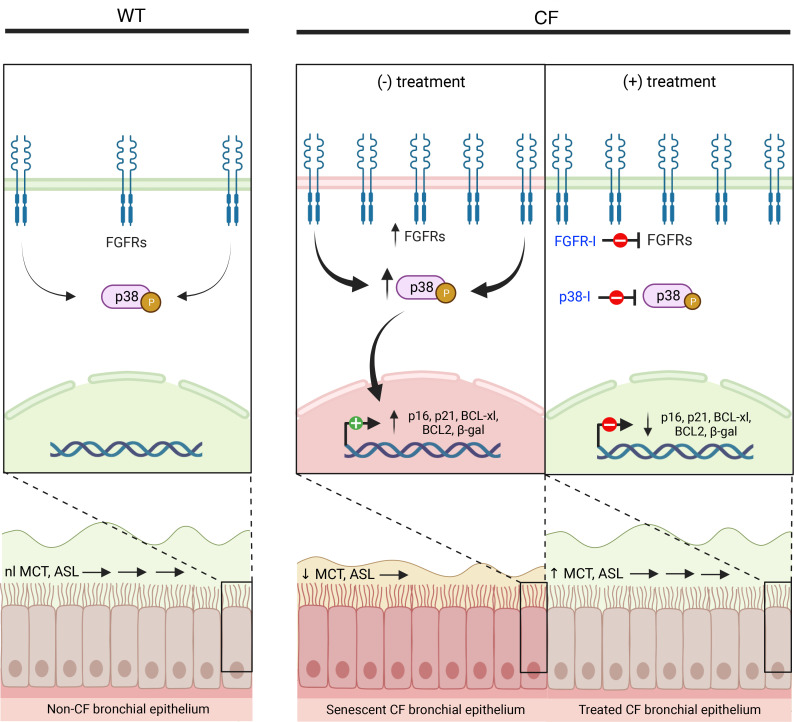

## Introduction

Cystic fibrosis (CF) is the most common autosomal recessive disorder, affecting more than 70,000 people worldwide (1). Respiratory failure is the leading cause of morbidity and mortality in people with CF (pwCF) (2). The emergence of highly effective modulator therapies (HEMTs) led to a significant decrease in disease burden and increased life expectancy, but chronic airway inflammation continues to persist, thereby affecting many cellular processes, leading to accelerated aging and lung function decline (3). Investigations of the aging biology in chronic lung diseases have advanced, and several cellular processes, termed “the hallmarks of aging,” have been used to characterize and study accelerated aging processes in lung diseases (4). However, little is known about the aging processes in the CF lung.

Cellular senescence is an aging hallmark defined by irreversible cell cycle growth arrest because of cellular stressors, like inflammation (5). Senescent cells are apoptosis resistant, have increased expression of senescence-associated β-gal (SA-β-gal), and develop a senescence-associated secretory phenotype (SASP) causing tissue damage, inflammation, and paracrine senescence (6). Molecular markers of senescence include B cell leukemia/lymphoma 2 (BCL2) and B cell lymphoma-extra-large (BCL-xL) for apoptotic resistance; cyclin-dependent kinase inhibitor 2A (p16) and cyclin-dependent kinase inhibitor 1 (p21) for cell cycle growth arrest; IL-6, IL-8, and IL-1β for SASP; and increased expression of SA-β-gal (7–9). Cellular senescence contributes to disease pathogenesis and progression in chronic obstructive pulmonary disease (COPD), idiopathic pulmonary fibrosis (IPF), and Alzheimer’s disease. Senolytic drugs targeting senescent cells in these diseases have proven to be beneficial in reversing disease course in preclinical models (10).

Fibroblast growth factor receptors (FGFRs) encompass a subfamily of receptor tyrosine kinases that consists of 4 family members (FGFR1–4) with diverse functions (11, 12). FGFR1 and -4 are increased in CF and COPD airways and regulate airway inflammation (13, 14). FGFR1 signaling contributes to airway inflammation in CF by activating the extracellular signal-regulated kinase/mitogen-activated protein kinase (ERK/MAPK) signaling cascade (13). FGFR4 induces airway inflammation through phospholipase C γ (PLCγ)/calcineurin and nuclear factor of activated T cells signaling (13, 14). FGFR signaling plays a complex role in cellular senescence with both pro- and antisenescence qualities (15–17). However, no studies to date have examined the consequences of cellular senescence and accelerated aging in the CF bronchial epithelium. To our knowledge, this is the first study to characterize cellular senescence in both in vitro and in vivo models of CF lung disease. We show that MAPK p38 signaling regulates cellular senescence in the CF bronchial epithelium and seems to involve FGFR4 and partially FGFR1, making FGFR blockade a novel and potential future amenable therapeutic target for senolytic therapies targeting the CF lung, which is independent of CFTR function.

## Results

### Single-cell RNA-sequencing data from primary CF airway epithelial cells demonstrate evidence of cellular senescence.

**Figure 1:**
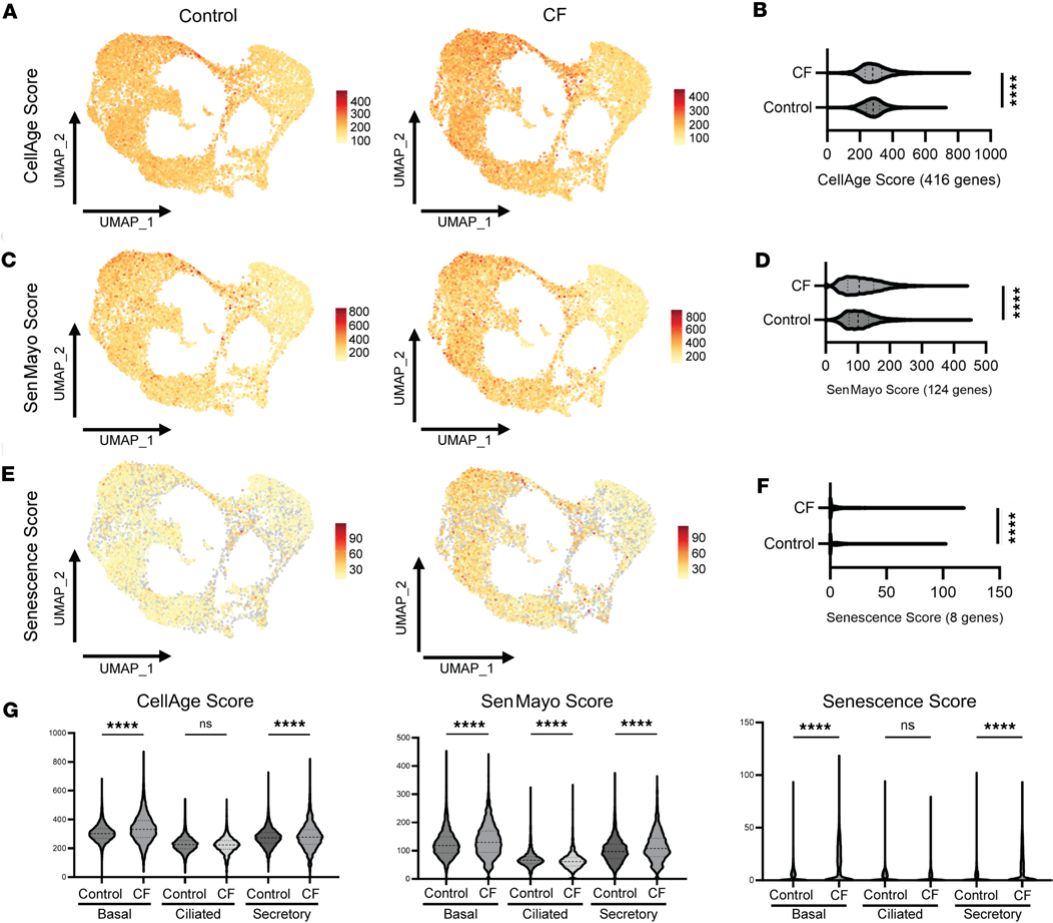
scRNA-Seq data reveal an increase in cell senescence markers in CF epithelial cells compared with control epithelial cells. Single-cell RNA transcriptome of control and CF epithelial cells (National Center for Biotechnology Gene Expression Omnibus GSE150674) from 19 control and 19 CF donor lungs from donors with end-stage CF lung disease and healthy controls, CF ΔF508 homozygous patients (*n* = 10,131 cells and *n* = 8 donors) and healthy donors (*n* = 23,119 cells and *n* = 19) were separated from the whole data set and analyzed using BBrowser3 to generate UMAPs and violin plots showing senescence scores from 3 separate gene databases: (**A** and **B**) CellAge database of senescence inducing genes (416 genes), (**C** and **D**) SenMayo (124 genes), and (**E** and **F**) cellular senescence markers used in this study (*CDKN1A*, *CDKN2A*, *BCL2*, *BCL2L1*, *IL6*, *IL1B*, *IL6*, and *GLB1*). (**G**) Senescence scores using the same 3 gene databases listed above but separated into violin plots looking at senescence scores based on major epithelial cell types: basal, ciliated, and secretory. Statistical analysis was done using unpaired Student’s *t* test or 1-way ANOVA shown with ****P < 0.0001.

To characterize cell senescence in the CF bronchial epithelium in a large, representative CF patient cell cohort, single-cell RNA sequencing (scRNA-Seq) data of airway epithelial cells from the previously published GSE150674 data set were used (18). This data set includes donors with end-stage CF lung disease and healthy controls and ages ranging 6–60 years in CF (*n* = 17,590 cells and *n* = 19 donors) and 18–63 years in healthy donors (*n* = 23,119 cells and *n* = 19 donors). Using uniform manifold approximation and projections (UMAPs), the data set was shown to contain a variety of cell types (Supplemental Figure 1A; supplemental material available online with this article; https://doi.org/10.1172/jci.insight.174888DS1), with the majority consisting of basal, ciliated, and secretory cells in both control and CF donors (Supplemental Figure 1B). For our analysis, we filtered the CF cell population to cells homozygous for the ΔF508 mutation (*n* = 10,131 cells and *n* = 8 donors with age range of 25–60 years) and created 3 senescence signature scores visualized via UMAPs and violin plot from 3 gene sets: CellAge senescence inducing genes (408 genes) database (19), SenMayo (124 genes) (20), and a commonly used cellular senescence marker panel (*CDKN1A*, *CDKN2A*, *BCL2*, *BCL2L1*, *IL6*, *IL1B*, and *GLB1*). UMAPs and violin plots examining the senescence score from the CellAge database showed a significant increase in the CF cohort compared with controls (Figure 1, A and B). Furthermore, the SenMayo score mean was significantly increased in the CF group compared with controls, with noticeable differences in the UMAP between CF and control groups (Figure 1, C and D). Moreover, we created a cellular senescence score using the following cellular senescence markers: *CDKN1A*, *CDKN2A*, *BCL2*, *BCL2L1*, *IL6*, *IL1B*, and *GLB1*, which demonstrated significant differences in the combined score visually and via violin plot in the CF epithelial cell group compared with control (Figure 1, E and F).

To identify the specific cell types involved, we subclassified the airway epithelial cells into the major representing groups and found significant differences in cellular senescence scores based on cell type. Using the CellAge database senescence score, basal and secretory epithelial cells from CF donors had a significantly increased score, when compared with non-CF controls, whereas there was no difference between ciliated cells from CF and control donors (Figure 1G). Similar results were seen using the SenMayo score (ciliated CF cells showed a decrease in senescence markers) (Figure 1G) and the 8-gene senescence score (Figure 1G). Overall, these data support the evidence of cellular senescence in the CF airway epithelium using multiple senescence scores with large data sets of genes associated with cellular senescence, which seems to be primarily localized to basal and secretory airway cells.

scRNA-Seq data revealed an increase in cell senescence in CF airway epithelial cells homozygous for the ΔF508 mutation.

To further characterize cellular senescence in GSE150674, we filtered the data set to compare senescence scores in homozygous ΔF508 cells, CF airway cells harboring other CF mutations, and control cells. Senescence scores in ΔF508 CF airway cells and non-∆508 CF airway cells were increased as shown via UMAPs, when compared with control (Supplemental Figure 2, A–C). Violin plots comparing the 3 groups showed a marked increase in all 3 senescence scores just in the ΔF508 CF airway cells (Supplemental Figure 2D). Overall, these data suggest that the CF airway cells from donors homozygous for the ΔF508 mutation exhibit increased senescence markers.

### Primary human bronchial epithelial cells from CF donors express increased cellular senescence markers.

**Figure 2:**
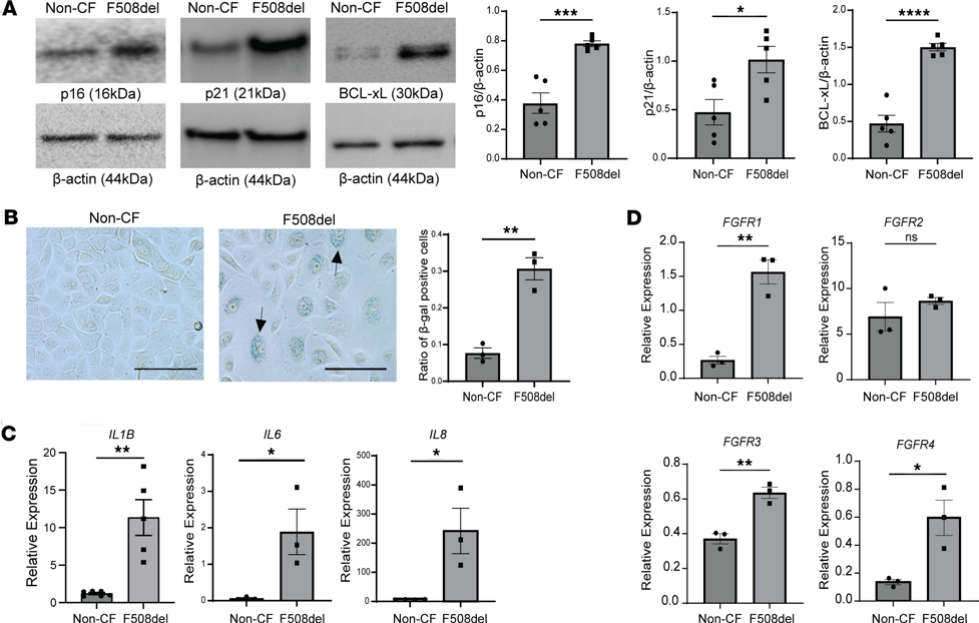
Cellular senescence markers are increased in CF primary human bronchial epithelial cells, cultured at the air liquid interface. (**A**) Representative immunoblot images and bar graphs showing densitometric analyses for p16, p21, and BCL-xL in CF ΔF508 and non-CF donors at ALI (*N* = 5). (**B**) Representative images for SA-β-gal staining using brightfield imaging of the same CF ΔF508 and non-CF donor ALI cultures including quantification of β-gal staining using the ratio of SA-β-gal–positive cells per brightfield by ImageJ (NIH) (*N* = 3); arrows show β-gal–positive cells (scale bar = 100 μm, original magnification, ×40). (**C**) Bar graphs demonstrating relative mRNA levels of SASP (*IL1B*, *IL6*, and *IL8*) markers normalized to GAPDH. (**D**) Bar graphs indicating relative mRNA levels of *FGFR1–4* normalized to GAPDH in the same 2 groups. Statistical analysis was done using unpaired Student’s *t* test showing means ± SEM with *P < 0.05, **P < 0.01, ***P < 0.001, and ****P < 0.0001 from 3–5 different donors per group with experiments repeated 3 times.

To validate the findings from the RNA-Seq data set, primary human bronchial epithelial cells, cultured at the air liquid interface (ALI) from donors with CF, who were homozygous for the ΔF508 mutation, and non-CF controls, were assessed for the expression of an established set of cell senescence markers (7). Similarly to the cells from the RNA-Seq data set, the differentiated primary CF ALI cultures also mainly consisted of basal cells, which were a higher percentage in non-CF donors (70%) versus CF donors (53%), with similar abundance of ciliated cells (18% non-CF versus 22% CF) and more secretory cells in the CF donor lungs, compared with the non-CF controls (25% versus 11%) (Supplemental Figure 3, A and B). Using Western blot imaging and densitometric analysis, we observed increases in p16, p21, and BCL-xL protein levels in the CF ALI cultures (Figure 2A). SA-β-gal staining was increased in CF ΔF508 ALI cultures, when compared with non-CF controls (Figure 2B). Additionally, mRNA levels of the SASP (*IL1B*, *IL6*, *IL8*) were also substantially increased in the CF ALI cultures (Figure 2C). Since we have previously shown an association between FGFR signaling and IL-8 secretion in the CF epithelium, including upregulation of FGFR1 (13), we compared the FGFR expression between control and CF ALI cultures. Interestingly, most FGFRs were markedly increased in CF ΔF508 primary human bronchial epithelial cells compared with non-CF control ALI cultures (Figure 2D). In summary, markers of cellular senescence and FGFR expression were increased in CF ΔF508 primary human bronchial epithelial cell ALI cultures, when compared with non-CF controls.

To further characterize cell type–specific localization of cell senescence markers, we colabeled ALI cultures with commonly used cell type–specific markers and senescence markers, which showed that secretory cells, which express uteroglobin (21), also showed expression of p16, p21, and BCL-xL (Supplemental Figure 4A and Supplemental Figure 9B). Colabeling of basal cells with KRT5 (22) exhibited less colocalization with p16 but overlapped with p21 and BCL-xL as well (Supplemental Figure 4B and Supplemental Figure 9B).

Furthermore, we attempted to validate these findings in human CF lung tissue sections and achieved labeling of secretory cells in sections of the bronchial epithelium, which colabeled with p16 (Supplemental Figure 5).

### FGFR inhibition decreases cellular senescence markers and phosphorylation of p38 MAPK.

**Figure 3:**
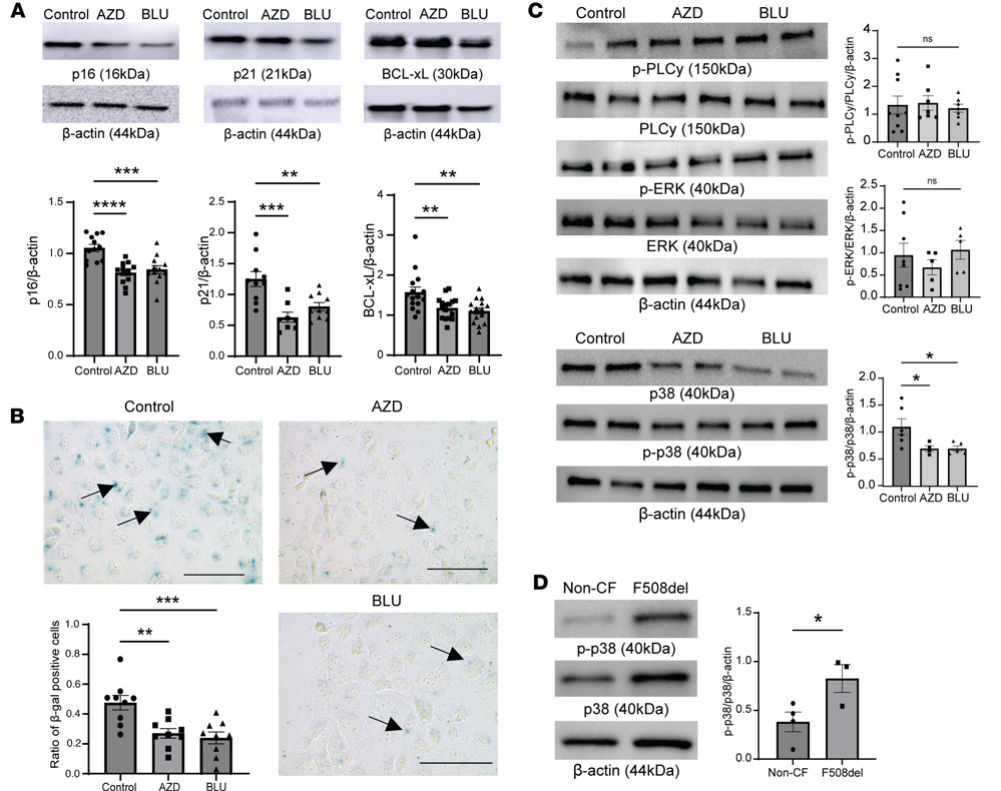
FGFR inhibition decreases cellular senescence markers and decreases phosphorylation of p38 MAPK. (**A**) Representative immunoblot images and densitometric analyses of the cellular senescence markers p16, p21, and BCL-xL from CFBEs, which were treated with AZD4547 0.1 μM or BLU9931 0.1 μM for 24 hours. (**B**) Representative images of SA-β-gal staining in CFBEs treated with AZD4547 and BLU9931 and quantification by capturing 3 images from different regions of the cell culture plates and counting total cells and β-gal–positive cells from the 3 images to make a ratio of β-gal–positive cells to total cells; arrows indicate β-gal–positive cells (scale bar = 100 μm, original magnification, ×40). (**C**) Representative immunoblots and densitometric analysis for p-ERK/ERK, p-PLC/PLCγ, and p-p38/p38 MAPK in CFBEs treated with AZD4547 and BLU9931 for 24 hours with β-actin loading control. (**D**) Representative immunoblot images and densitometric analyses from primary bronchial epithelial ALI cultures of CF (ΔF508) donors and non-CF control donors for p-p38/p38 MAPK expression. Statistical analysis was done using unpaired Student’s *t* test showing means ± SEM with *P < 0.05, **P < 0.01, and ***P < 0.001; 3 independent experiments were done in triplicates.

CF bronchial epithelial cells (CFBEs) were treated with different FGFR inhibitors, including the clinically used FGFR1–3 inhibitor AZD4547 (13, 23–25) and the FGFR4 inhibitor BLU9931 (26). Expression of p16, p21, and BCL-xL showed a marked attenuation following treatment with each specific inhibitor (Figure 3A). Furthermore, the ratio of SA-β-gal–positive cells in the CFBEs treated with AZD4547 or BLU9931 was substantially lower compared with vehicle-treated cells (Figure 3B). Next, we examined the activation of the downstream signaling mediators of FGFRs, including ERK, PLCγ, and p38 MAPK (13, 14, 27–30). CFBEs, treated for 24 hours with AZD4547 or BLU9931, did not show any relevant differences in phosphorylation of PLCγ or ERK (Figure 3C). However, there was a marked decrease in p38 MAPK phosphorylation in CFBEs treated with AZD4547 or BLU9931 (Figure 3C). Primary human airway CF ALI cultures exhibited a baseline increase in p38 MAPK phosphorylation compared with non-CF controls (Figure 3D).

BLU9931 is a potent, selective, and irreversible FGFR4 inhibitor with over 50-fold selectivity over FGFR1–3 (16), whereas the clinically established and used inhibitor AZD4547 has selectivity for FGFR1–3 at very similar concentrations. In addition, AZD4547 demonstrates weaker activity against FGFR4, VEFGR2, and p38. To further characterize the isoform-specific FGFR responsible for the demonstrated “senolytic” effects, we treated CF ALI cultures with PD173074, a potent and more selective FGFR1 inhibitor (IC_50_ of 25 nM), at 25 and 50 nM (31, 32).

Targeting FGFRs via siRNA knockdown, specifically in ALI cultures, is challenging and will not block residual kinase activity; therefore, inhibitors have been shown to work best for our in vitro studies (13, 14). PD173074-treated CF ALI cultures showed a dose-dependent reduction in p16 expression (Supplemental Figure 6A) but no changes in p21 and BCL-xL expression (Supplemental Figure 6, B and C).

Assessment of the SASP markers IL-6 and IL-8 via ELISA from basolateral media also demonstrated a decrease in PD173074-treated cells, which was in a dose-dependent manner for IL-8 (Supplemental Figure 6D). Interestingly, AZD4547 and BLU9931 treatment did not affect IL-6 and -8 levels in the basolateral media of CF ALI cultures (Supplemental Figure 7, A and B). In summary, FGFR inhibition attenuated cellular senescence in the CFBEs, which seems to be mediated by FGFR signaling, pointing to partial dependence on FGFR1 and a more comprehensive involvement of FGFR4 in vitro.

### Inhibition of p38 MAPK decreases cellular senescence markers in CFBEs.

**Figure 4:**
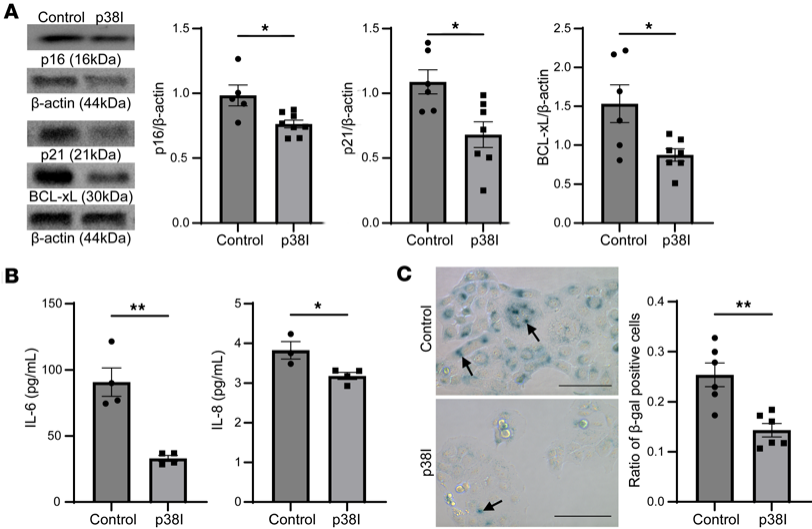
Inhibition of p38 MAPK decreases cellular senescence markers in CFBEs. (**A**) Representative immunoblot images and densitometric analyses showing p16, p21, and BCL-xL expression of CFBEs treated with SB203580 at 20 μM for 24 hours compared with controls. (**B**) Bar graphs showing protein levels of IL-6 and IL-8 in CFBE supernatant after treatment with SB203580 for 24 hours. (**C**) Representative images of SA-β-gal staining in control and SB203580-treated CFBEs including quantification; arrows indicate β-gal–positive cells (scale bar = 100 μm, original magnification, ×40). Statistical analysis was done using unpaired Student’s *t* test showing means ± SEM with *P < 0.05, **P < 0.01 with *n* = 3–5 experiments.

To further investigate whether p38/MAPK mediates cellular senescence in the CF bronchial epithelium, CFBEs were treated with a p38/MAPK inhibitor (SB203580) for 24 hours. A marked decrease in protein expression of p16, p21, and BCL-xL was observed following pharmacological blockade of p38/MAPK (Figure 4A). In addition, the SASP cytokines IL-6 and IL-8 were also attenuated by p38/MAPK inhibition in CFBEs (Figure 4B). These findings were also accompanied by a considerable decrease in the ratio of SA-β-gal–positive cells in CFBEs treated with the p38 MAPK inhibitor (Figure 4C). Together, these data suggest that multiple cellular senescence markers are regulated by p38 MAPK in the CF bronchial epithelium.

### Cellular senescence markers are increased in lung tissue and the airway epithelium of Cftr^–/–^ rats compared with littermate controls.

**Figure 5:**
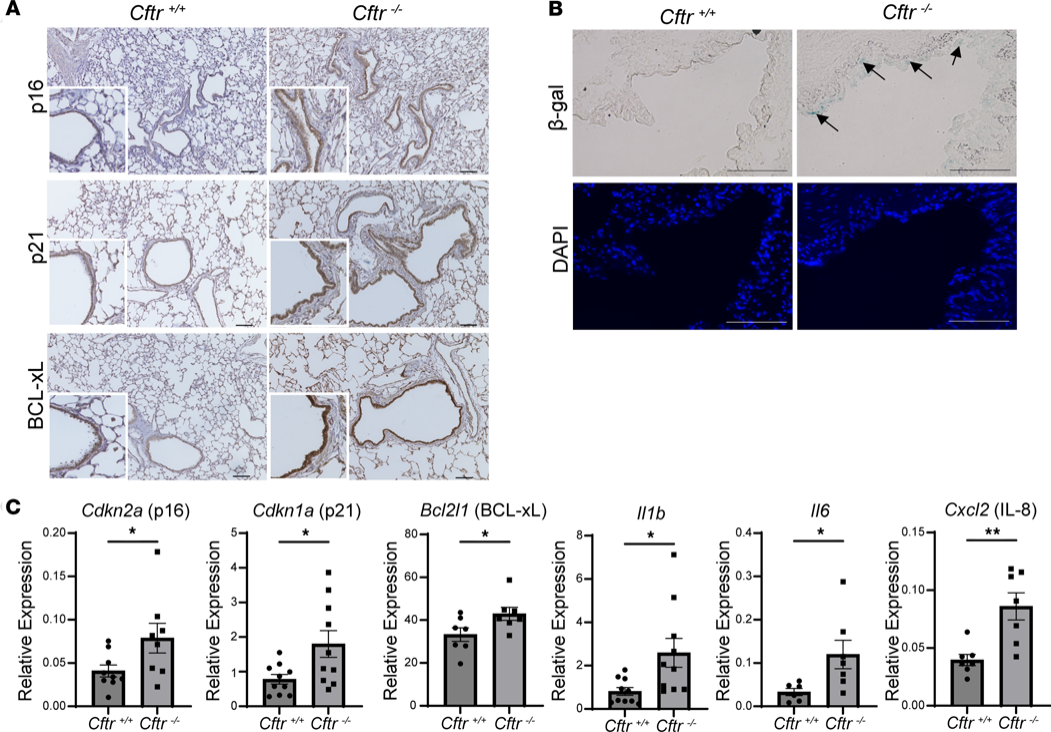
Cellular senescence markers are increased in 6-month-old *Cftr^–/–^* rat lungs compared with controls. (**A**) Immunohistochemical staining for p16, p21, and BCL-xL in *Cftr^–/–^* rat lung tissue compared to controls demonstrating an increased signal in the bronchial epithelium (scale bar = 100 μm, original magnification, ×10; for insets, ×20). (**B**) SA-β-gal stain and nuclear counterstain (DAPI) in lung tissue from *Cftr^–/–^* rats and littermate controls; arrows indicate areas of airway epithelial β-gal staining (scale bar = 100 μm, original magnification, ×40). (**C**) Relative mRNA levels of *Cdkn2a* (p16), *Cdkn1a* (p21), *Bcl2l1*, and SASP markers (*Il1b*, *Il6*, and *Cxcl2* [IL-8]) normalized to GAPDH, from total lung tissue of control and *Cftr^–/–^* rats. Statistical analysis was done using unpaired Student’s *t* test showing means ± SEM with *P < 0.05, **P < 0.01 with *n* = 5–10 rats per group and experiments done in triplicates.

**Figure 6:**
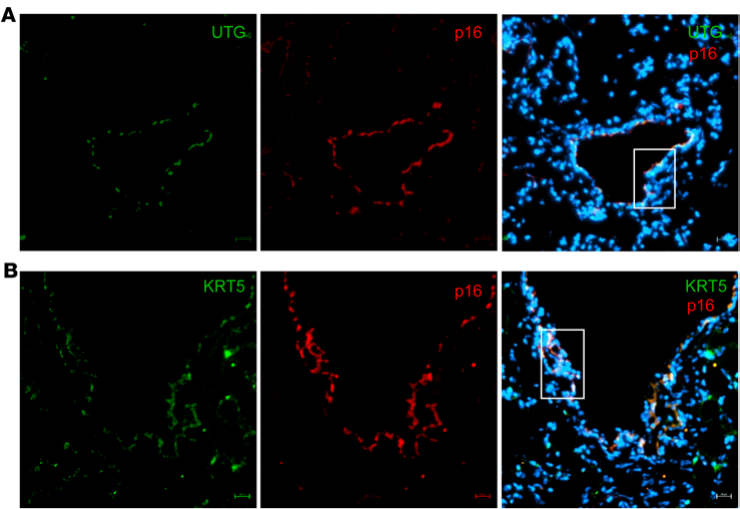
Expression of p16 in both basal cells and secretory airway epithelial cells in 6-month-old *Cftr^–/–^* rat lungs. (**A** and **B**) Immunofluorescence staining for p16 and colabeled with either Krt5 (KRT5; secretory cells) or uteroglobin (UTG; basal cells) in *Cftr^–/–^* rat lung tissue and nuclear staining with DAPI. Boxes indicate bronchial epithelium that shows the double labeling. The scale bar is 20 µm, and it is a ×40 original magnification.

Six-month-old *Cftr^–/–^* rats, a model that exhibits CF-like airway disease (33), were used to validate our findings of increased cellular senescence in vivo. Lung tissue from 6-month-old *Cftr^–/–^* rats and controls was assessed via immunohistochemistry and showed increased staining of p16, p21, and BCL-xL in the bronchial epithelium (Figure 5A), when compared with littermate controls and secondary antibody control only (Supplemental Figure 8, A and B). Furthermore, SA-β-gal staining was increased in the *Cftr^–/–^* rat lungs compared with controls (Figure 5B). mRNA levels for *Cdkn2a* (p16), *Cdkn1a* (p21), *Bcl2l1* (*BCL-xL*), and SASP markers (*Il-1b*, *Il-6*, *Cxcl2*) from total lung tissue were also significantly increased in comparison with *Cftr*^+/+^ lungs (Figure 5C). To show bronchial localization of some of the senescence markers, we used immunofluorescence staining and colabeled frozen sections of *Cftr^–/–^* rat lung tissue, demonstrating colocalization of p16 with uteroglobin, a secretory cell marker, and KRT5, a basal cell marker, validating our in vitro results (Figure 6, A and B). KRT5, uteroglobin, and p16 staining was validated by comparison with respective secondary antibody–only controls (Supplemental Figure 9A). In summary, there is evidence of cellular senescence in the lung and bronchial epithelium from a well-established in vivo model exhibiting CF airway disease.

### FGFR expression was increased in the Cftr^–/–^ rat lung.

**Figure 7:**
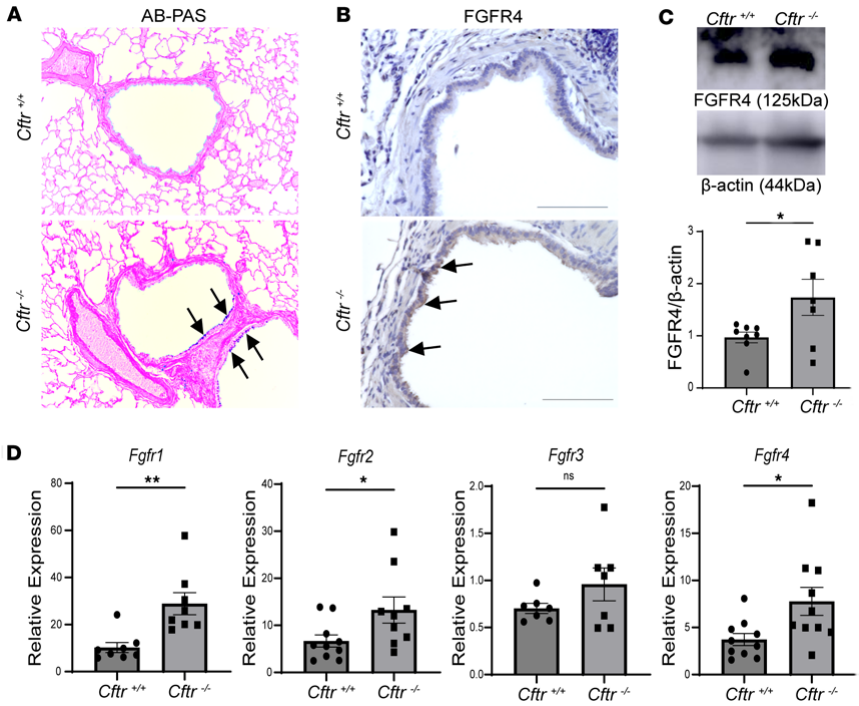
FGFR expression is increased in the lungs of 6-month-old *Cftr^–/–^* rats. (**A**) AB-PAS staining indicating an increase of intercellular mucus staining in the bronchial epithelium of *Cftr^–/–^* rats, compared with control rats; arrows show areas stained in blue for intercellular mucus (original magnification, ×20). (**B**) Immunohistochemical analysis using an isoform-specific and validated anti-FGFR4 showed increased staining in the bronchial epithelium of *Cftr^–/–^* rats; arrows highlight areas of airway epithelium stained for FGFR4 (scale bar = 100 μm, original magnification, ×40). (**C**) Representative images of FGFR4 and β-actin with densitometric analysis demonstrating increased FGFR4 expression in *Cftr^–/–^* rat airways. (**D**) Relative mRNA levels of *Fgfr1–4* from *Cftr^–/–^* total lung tissue, normalized to GAPDH expression. Statistical analysis was done using unpaired Student’s *t* test showing means ± SEM with *P < 0.05, **P < 0.01 with *n* = 5–10 rats per group.

Formalin-fixed, paraffin-embedded sections of total lung tissue from 6 month-old *Cftr^–/–^* rats were stained with Alcian blue–periodic acid–Schiff (AB-PAS) to recapitulate the previously established muco-obstructive phenotype when compared with controls (Figure 7A) (33). Immunohistochemical analysis using an isoform-specific antibody against FGFR4 revealed increased staining of the *Cftr^–/–^* bronchial epithelium, when compared with airways from wild-type littermates (Figure 7B). FGFR4 protein expression, determined by Western blot analysis, was also substantially increased in *Cftr^–/–^* rat lungs when compared with controls (Figure 7C). A dearth of validated antibodies limited our ability to assess protein expression of FGFR1–3, but we have previously shown that FGFR protein expression correlated with mRNA levels (14); therefore, quantitative real-time PCR was performed and validated a marked increase in mRNA levels of *Fgfr1*, *Fgfr2*, and *Fgfr4* in *Cftr^–/–^* rat lung tissue (Figure 7D). Transcript levels of the FGFRs have been shown previously to corroborate with protein expression (14). In summary, cell senescence markers as well as FGFRs are upregulated in the lungs of 6-month-old *Cftr^–/–^* rats.

### Treatment of Cftr^–/–^ rats with the FGFR inhibitor AZD4547 attenuates cell senescence in the lungs and bronchial epithelium, which correlates with marked improvements in mucociliary clearance.

**Figure 8:**
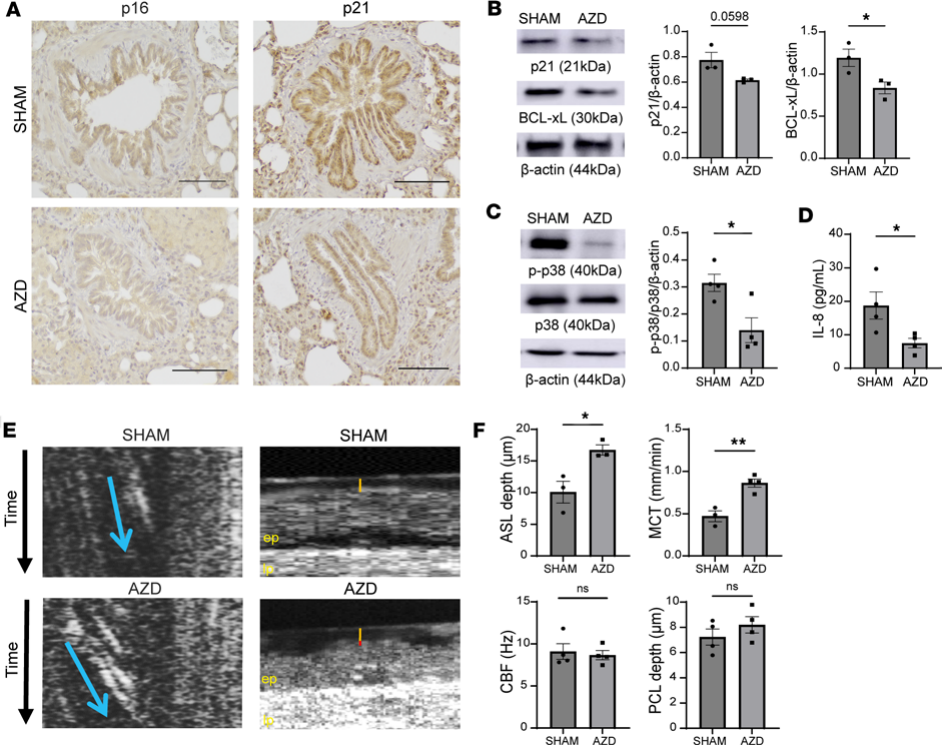
Systemic FGFR inhibition in *Cftr^–/–^* rats leads to decreased cellular senescence in the lung and improved mucociliary clearance. (**A**) Representative images of immunohistochemical staining for p16 and p21 in *Cftr^–/–^* and control rat lungs ± AZD4547 treatment (scale bar = 100 μm, original magnification, ×20). (**B**) Representative immunoblot images and bar graphs demonstrating densitometric analyses of p21 and BCL-xL protein expression in *Cftr^–/–^* rat lungs ± AZD4547 treatment. (**C**) Representative immunoblot images of phosphorylated and total p38 MAPK and densitometric analysis. (**D**) IL-8 protein levels in *Cftr^–/–^* rat lung tissue ± AZD4547 treatment. (**E**) Representative images showing mucociliary transport (MCT) (cross-sectional arrow in blue indicates the velocity of the mucus particle via the slope), along with representative µOCT images of the trachea of *Cftr^–/–^* rats (yellow line representing airway surface liquid [ASL] depth and the red line representing periciliary liquid depth; ep, epithelial layer; lp, lamina propria). (**F**) Bar graphs indicating analysis of µOCT images quantifying ASL, ciliary beat frequency (CBF), periciliary liquid depth (PCL), and MCT from *Cftr^–/–^* rat trachea after treatment for 5 days with AZD4547 (12.5 mg/kg) or sham. Statistical analysis was done using unpaired Student’s *t* test showing means ± SEM with *P < 0.05, **P < 0.01, with *n* = 3–4 rats per group.

To investigate the effects of FGFR inhibition on “reversal” of cellular senescence in vivo, *Cftr^–/–^*rats were treated with AZD4547 via oral gavage daily for a total of 5 days. Pharmacological blockade of FGFRs with AZD4547 led to a decrease in p16 and p21 staining in the bronchial epithelium of *Cftr^–/–^* rats compared with sham-treated *Cftr^–/–^* rats (Figure 8A). Furthermore, total lung protein expression of p21 and BCL-xL from AZD4547-treated *Cftr^–/–^* rats was significantly decreased when compared with sham-treated rats (Figure 8B). A reduction in phosphorylation of p38 MAPK expression (Figure 8C) and IL-8 secretion (Figure 8D) was also observed. Tracheae of AZD4547-treated *Cftr^–/–^* rats were analyzed via micro-optical coherence tomography (μOCT) (Figure 8, E and F), demonstrating marked improvements in ASL depth but no considerable differences in CBF or PCL depth, suggesting treatment with AZD4547 did not negatively affect the functional microanatomy of the lung epithelium (Figure 8, E and F). Further, there were substantial improvements in MCT in the AZD4547-treated *Cftr^–/–^* rat trachea when compared with the vehicle treatment (Figure 8F). Overall, these data validate our in vitro findings that there is a decrease in cell senescence markers after FGFR inhibition with functional consequences, leading to improved mucociliary clearance without affecting the microanatomy of the CF airway epithelium.

### Isoform-specific inhibition of FGFR4 in an ex vivo Cftr^–/–^ rat trachea model decreases cellular senescence and partially restores mucociliary clearance.

**Figure 9:**
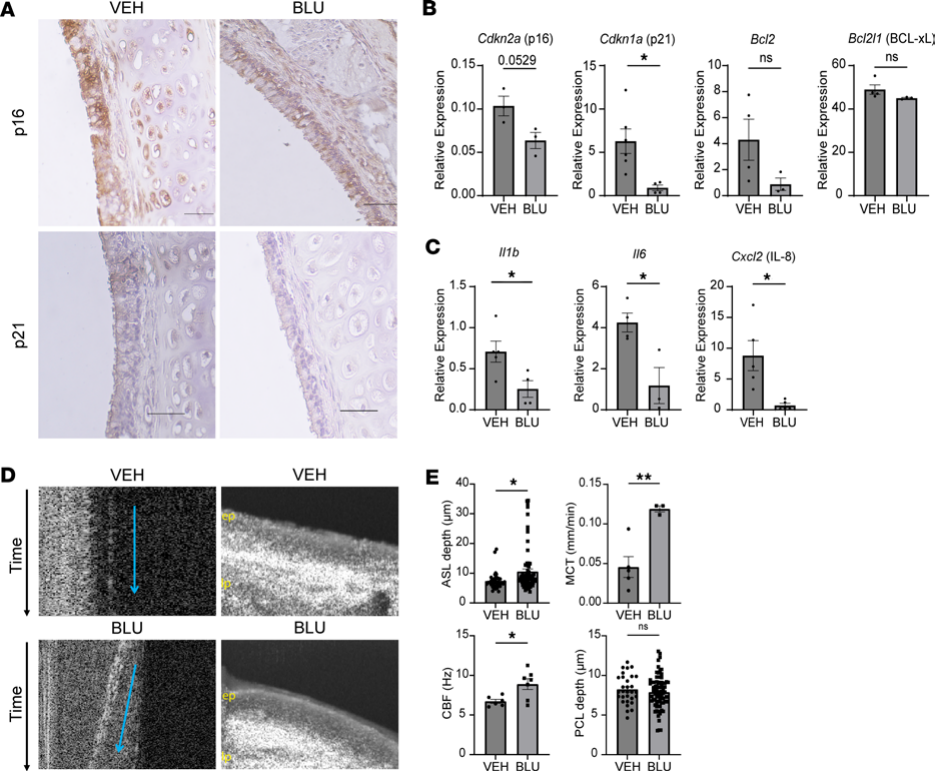
Isoform-specific FGFR4 inhibition in an ex vivo *Cftr^–/–^* rat trachea model improves mucociliary clearance with attenuation of senescence markers. (**A**) Immunohistochemistry of p16 and p21 from ex vivo *Cftr^–/–^* rat tracheae treated with BLU9931 at 0.1 M for 24 hours compared with vehicle-treated “control” *Cftr^–/–^* rat tracheae. (**B**) mRNA levels of *Cdkn2a* (p16), *Cdkn1a* (p21), *Bcl2*, and *Bcl2l1* (BCL-xL) and SASP markers (**C**) *Il1b*, *Il6*, and *Cxcl2* (IL8) from the ex vivo *Cftr^–/–^* rat tracheae +/– BLU9931. (**D**) Representative µOCT images (ep, epithelial layer; lp, lamina propria) and (**E**) bar graphs showing µOCT quantification of periciliary liquid depth (PCL), mucociliary transport (MCT), ciliary beat frequency (CBF), and airway surface liquid (ASL) depth both from BLU9931 and vehicle control treated ex vivo *Cftr^–/–^* rat tracheae. Statistical analysis was done using unpaired Student’s *t* test showing means ± SEM with *P < 0.05, **P < 0.01, with *n* = 3–4 rat trachea per group.

To validate our in vitro findings that FGFR4 inhibition attenuated cell senescence and assess functional outcomes, we used an ex vivo *Cftr^–/–^* rat trachea model (34) and treated with BLU9931 (0.1 μM for 24 hours) (Figure 9). BLU9931 treatment decreased p16 and p21 levels shown through immunohistochemistry staining of ex vivo *Cftr^–/–^* rat tracheae compared with the vehicle and secondary only controls (Figure 9A and Supplemental Figure 8C). In addition, *Cdkn2a* (p16), *Cdkn1a* (p21), *Bcl2*, and *Bcl2l1* (BCL-xL) (Figure 9B) and SASP marker (*Il1b*, *Il6*, *Cxcl2* [IL-8]) (Figure 9C) mRNA levels were decreased in BLU9931-treated *Cftr^–/–^* rat tracheae, when compared with vehicle-treated *Cftr^–/–^* tracheae. Treatment with BLU993 also led to marked improvements in mucociliary clearance, including restoration of MCT and increased ASL depth as well as improved CBF without changes in PCL depth (Figure 9, D and E). In summary, these data imply that isoform-specific inhibition of *Fgfr4* in an ex vivo CF model led to restoration of mucociliary clearance, which was accompanied by attenuation of several cell senescence markers.

### Treatment with dasatinib and quercetin decreases cellular senescence and improves mucociliary clearance in the ex vivo Cftr^–/–^ rat trachea model.

**Figure 10:**
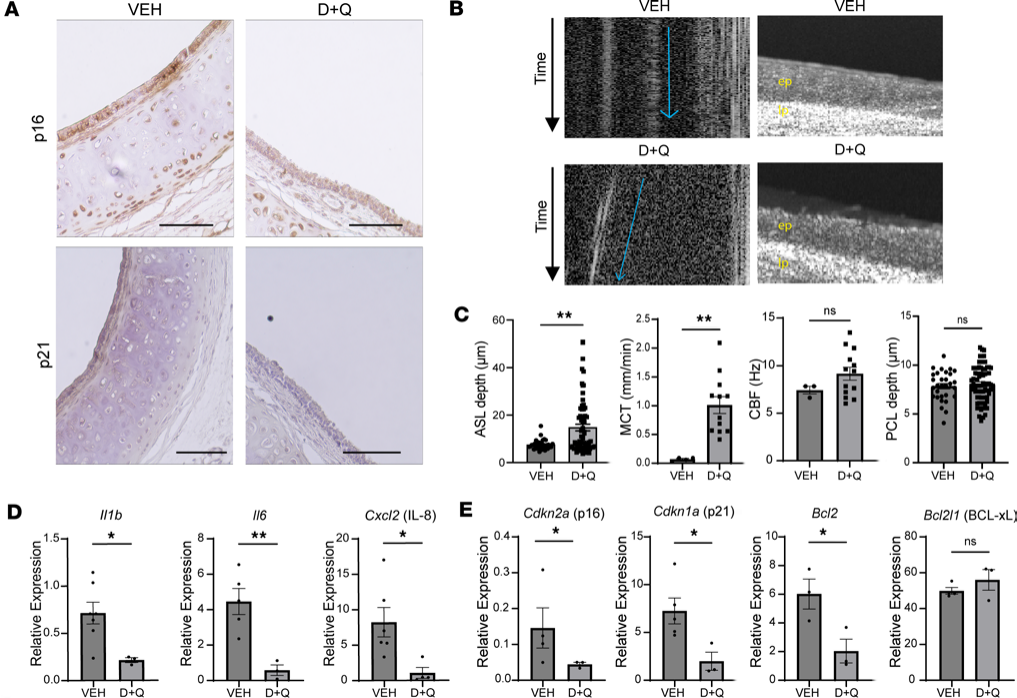
Treatment with dasatinib and quercetin significantly decreases cellular senescence and improves mucociliary clearance in the ex vivo *Cftr^–/–^* rat trachea model. (**A**) Immunohistochemistry of *Cdkn2a* (p16) and *Cdkn1a* (p21) of *Cftr^–/–^* rat tracheae, which were treated with 100 nM dasatinib and 2 μM quercetin (D+Q) for 24 hours, compared with vehicle-treated *Cftr^–/–^* rat tracheae. Scale bar is 100 µm. (**B**) Representative µOCT images of the different *Cftr^–/–^* rat trachea groups (ep, epithelial layer; lp, lamina propria), including representative images to assess mucociliary transport (MCT). (**C**) Bar graphs showing quantification of all regions of interest from µOCT images for assessment of ASL, MCT, CBF, and PCL from the vehicle- and D+Q-treated groups. (**D**) mRNA levels of SASP markers (*Il1b*, *Il6*, and *Cxcl2* [IL-8]) along with (**E**) senescence markers *Cdkn2a* (p16), *Cdkn1a* (p21), *Bcl2*, and *Bcl2l1* (BCL-xL) from *Cftr^–/–^* rat tracheae ± D+Q. Statistical analysis was done using unpaired Student’s *t* test showing means ± SEM with *P < 0.05, **P < 0.01 with *n* = 3–4 rat tracheae per group.

To test whether cellular senescence itself contributes to mucociliary dysfunction, we utilized a widely used combination of senolytic drugs (dasatinib and quercetin: D+Q), which have been shown to attenuate cell senescence in other cell types, including the lung (35, 36), and treated *Cftr^–/–^* rat tracheae with D+Q for 24 hours. As shown previously in other systems, D+Q treatment caused a reduction in p16 and p21 compared with vehicle and secondary only controls (Figure 10A and Supplemental Figure 8C). µOCT assessment of both trachea groups showed that D+Q treatment also led to restoration of MCT and ASL depth without affecting CBF and PCL depth (Figure 10, B and C). Furthermore, *Il1b*, *Il-6*, and *Cxcl2* mRNA levels were also substantially decreased in D+Q-treated *Cftr^–/–^* tracheae, when compared with vehicle-treated *Cftr^–/–^* tracheae (Figure 10D). *Cdkn2a* (p16), *Cdkn1a* (p21), and *Bcl2* mRNA levels were also downregulated with D+Q treatment (Figure 10E). In summary, targeting senescence in the CF airway ex vivo using senolytic therapy improves mucociliary clearance.

### HEMT does not substantially decrease cellular senescence markers in CF airways.

**Figure 11:**
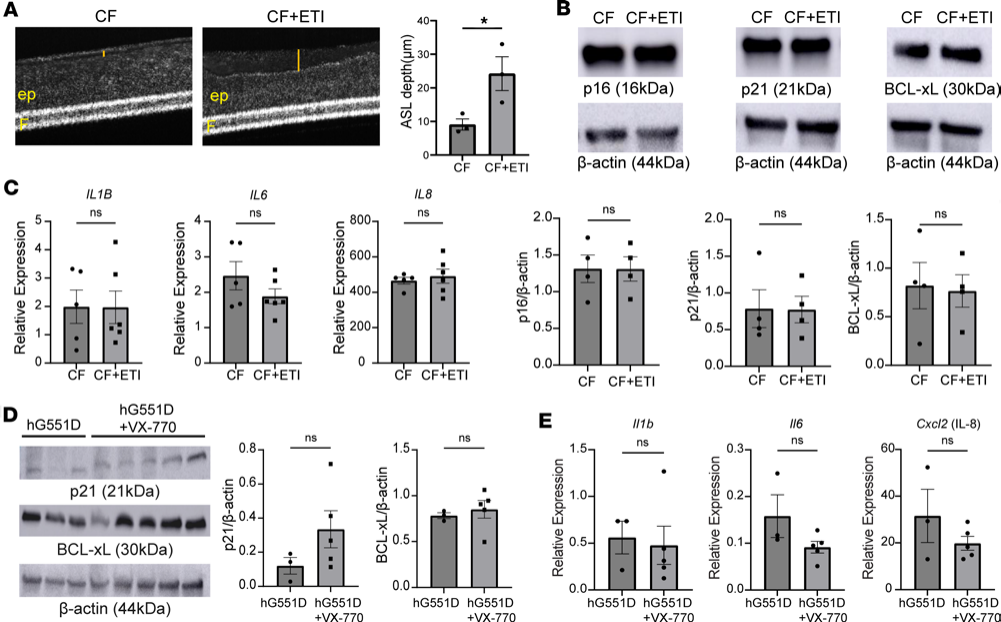
HEMT does not significantly decrease cellular senescence markers or expression of FGFRs. (**A**) Representative µOCT images and bar graph of CF primary human bronchial epithelial cells (*n* = 3 donors per group) treated with VX-661/VX-445/VX-770 (ETI) for 72 hours showing a significant increase in ASL depth in CF cells treated with ETI; yellow line is ASL depth. ep, epithelial layer; F, filter. (**B**) Representative immunoblots and densitometric analysis showing no significant difference in protein expression of cellular senescence markers p16, p21, and BCL-xL in CF primary human bronchial epithelial cells treated with ETI for 72 hours when compared with untreated CF primary human bronchial epithelial cells (*n* = 4 donors per group). (**C**) Relative expression of SASP markers (*Il1b*, *Il6*, and *Cxcl2* [IL-8]) from CF primary human bronchial epithelial cells demonstrates no significant change when compared with untreated CF primary human bronchial epithelial cells. (**D**) Immunoblots and densitometric analysis of p21 and BCL-xL in hG551D rats and hG551D rats treated with VX-770 for 14 days (hG551d = 3 rats, hG551d+VX-770 = 5 rats). (**E**) Relative expression of SASP markers *Il1b*, *Il6*, and *Cxcl2* (IL-8) in hG551D rats and hG551D rats treated with VX-770 for 14 days (hG551d = 3 rats, hG551d+VX-770 = 5 rats). Statistical analysis was done using unpaired Student’s *t* test showing means ± SEM with *P < 0.05.

To investigate whether HEMT could affect cellular senescence, we treated CF primary human bronchial epithelial cells on ALI with VX-661/VX-445/VX-770 (ETI) and vehicle. First, we demonstrated ETI corrected *CFTR* dysfunction by μOCT analysis of ASL depth in CF primary bronchial epithelial cells showing ASL depth restoration (Figure 11A). However, we did not find any difference in protein expression of the cellular senescence markers p16, p21, and BCL-xL (Figure 11B). To further define the effect of ETI on cellular senescence, we examined relative expression of SASP markers and found that there was no difference in relative expression between ETI-treated and vehicle-treated groups either (Figure 11C). Additionally, we did not see differences in *CDKN2a* (p16), *CDKN1a* (p21), or *BCL2* with ETI (Supplemental Figure 10, A–C), nor did the combination of ETI + FGFR inhibition improve these markers (Supplemental Figure 10, A–F). FGFR expression was also not different between groups (Supplemental Figure 11A). Moreover, we assessed cellular senescence in an hG551D rat model, which is receptive to ivacaftor (VX-770) treatment (37). Immunoblots from hG551D rat lungs treated with VX-770 demonstrated no considerable changes in protein levels of the cellular senescence markers p21 and BCL-xL (Figure 11D) or SASP markers (Figure 11E). There was also no difference noted in expression of the different *Fgfr* isoforms (Supplemental Figure 11B). Furthermore, the hG551D rat model itself did not exhibit any increase in cell senescence in its lung, when compared to wild-type littermates (Supplemental Figure 12, A–E). Taken together, these results show that CFTR correction in CF did not attenuate cellular senescence in our in vitro and in vivo models.

## Discussion

In this study, to our knowledge, we are the first to demonstrate evidence of increased cellular senescence in both in vitro and in vivo CF models, which was paralleled by an increase in FGFR expression. Pharmacological inhibition of FGFRs led to a decrease in cell senescence, which seemed to be at least partially mediated by MAPK p38. The direct role of the FGFR/MAPK p38 signaling axis on cell senescence was also validated in an in vivo CF rat model. Furthermore, pharmacological inhibition of the FGFRs altered mucociliary clearance and ASL volume, 2 important functional outcomes of CF airway disease severity (38). Additionally, using scRNA-Seq data (GSE150674), we were able to validate cellular senescence as a feature of CF airway epithelial cells (Figure 1), mainly of basal and secretory airway cells, including more comprehensive cell senescence marker panels, which have been useful in studying pathobiology in other lung diseases, such as IPF (39).

Cellular senescence was first discovered by Hayflick in the 1960s and has since been described as a hallmark of aging and a common feature found in disease and age-associated diseases (40). Several studies have shown that removal of senescent cells via senolytic treatments can reduce the number of senescent cells and significantly decrease disease burden in other chronic lung diseases (10, 41). Our study suggests that FGFR inhibition may attenuate expression of several cell senescence markers, which could be transiently expressed in the CF bronchial epithelium or be a sign of dysfunctional repair as has been shown in the murine alveolar epithelium, when injured (42). Furthermore, our in vitro data do not point to highly effective CFTR modulator therapy attenuating cell senescence markers in primary ALI homozygous for the ΔF508 mutation, nor were there changes in the hg551D rats that were treated with ivacaftor (Figure 11 and Supplemental Figures 10–12). Nevertheless, those treatments were short-term, and there might be a benefit in patients, who have been on HEMT long-term, which needs future investigation. In addition, cell senescence markers were predominantly upregulated in the ΔF508 CFBEs, but we did not analyze a sufficient number of G551D donors or donors with nonsense mutations to draw conclusions. However, hg551D rats did not show increased senescence where cell senescence was observed in *Cftr^–/–^* rat airways.

FGFR signaling plays a complex role in cellular senescence, and there is evidence for both pro- and antisenescence effects. For example, FGFR signaling has been shown to induce cellular senescence in pancreatic cancer cells (7, 16); however, FGFRs have been shown to delay or prevent senescence in stem cells, fibroblasts, and neurons (15, 17, 43). Additionally, FGFR signaling has been shown to regulate telomerase activity, which prevents telomere shortening and reduces cellular senescence (44).

FGFR signaling has been studied extensively by our lab and others in multiple organs, including the lung, kidney, heart, and parathyroid glands (13, 14, 27–30). FGFR1 signaling can occur via binding to FGF23 and α-klotho, leading to activation of ERK. FGFR4 has been shown to be abundantly expressed in the lung and the bronchial epithelium and shows activation and downstream signaling via PLCγ phosphorylation in the COPD lung (14). In our study, all FGFRs were expressed in the bronchial epithelium, which mainly consisted of basal epithelial cells and secretory cells. Those cell types also showed consistent expression of cell senescence markers, which were increased in the CF donors. Interestingly, the RNA-Seq data set also showed upregulation of FGFRs in the ΔF508 CF data set, which was also shown when data were combined with the senescence scores (Supplemental Figures 13 and 14). However, there was relatively low expression of FGFR4, which was abundantly expressed and upregulated in the CF ALI cultures, and PLCγ phosphorylation was not increased in CFBEs (Figure 3C). Furthermore, FGFR3 was not consistently upregulated in the CF rat lung (Figure 7D). In previous reports, FGFR3 expression was associated with lung cancer (45), whereas FGFR2 played an important role in alveolar epithelial cell homeostasis and survival following injury (46, 47). A previous study found that FGFR1 plays a role in *Cftr* maturation (36). However, to date no data suggest FGFR regulation of cell senescence in the CF lung.

Our results suggest that FGFR inhibition leads to a reduction in cell senescence, which seems to be partially mediated by MAPK p38 Given that cellular senescence is defined by various markers and involves intricate crosstalk of signaling pathways, FGFR signaling may overlap with upstream/downstream signaling molecules, causing FGFR compensatory mechanisms (48–50). In addition, tools to target isoform-specific FGFRs are limited, with FGFR1-knockout mice not being viable and siRNA-mediated FGFR knockdown not being efficient because of residual tyrosine kinase activity. Therefore, we used a pharmacological approach against FGFR isoforms that has limitations, including potential lack of isoform specificity and off-target effects. We have shown previously that α-klotho in the CF airways exhibits an antiinflammatory action and can attenuate FGF23- and TGF-β–mediated IL-8 secretion (13). However, other studies have shown that FGFR inhibition can regulate downstream mediators without a ligand (51). In order to specify which FGFR isoform mainly contributes to mediation of cell senescence, we used isoform-specific FGFR inhibitors. Some of these FGFR inhibitors are used in clinical studies. Targeting FGFR1 and FGFR4 as specifically as possible, we could demonstrate that both receptors seem to be involved in the regulation of cell senescence markers, but FGFR4 inhibition showed attenuation of cell senescence in a more comprehensive manner (Figure 9). Interestingly, when using AZD4547, which can inhibit not only FGFR1 but also FGFR2 and -3 and other tyrosine kinases, we saw a greater response when compared with a more specific FGFR1 inhibitor (Figure 3 and Supplemental Figure 6). Those results show that FGFR signaling is complex and receptor isoforms might be able to compensate for each other, which makes validation of those inhibitors also quite challenging in in vitro systems that do express all 4 FGFRs. In addition, many of the cell senescence markers are target genes of NF-κB signaling, but we observed neither any phosphorylation of p65 in vivo and in vitro nor FGFR inhibition affecting phosphorylation of p65 (Supplemental Figure 15). Further studies are needed to investigate signaling pathways involved as well as upstream signaling regarding which FGF ligands might contribute. The *Fgfr4*-knockout mouse is not lethal, though we have shown that the adult mouse develops airway inflammation along with changes consistent with emphysema; the murine lung is not a great model to study airway biology, especially CF (27, 52). In addition, FGFR4 inhibition has been studied in cancer, though whether FGFR4 is a suitable target in cancer therapy is still controversial (53). Future studies could include neutralizing isoform-specific FGFR antibodies to avoid off target effects.

Furthermore, whether cell senescence is mediating functional outcomes in the CF bronchial epithelium is of interest, and our studies potentially show an indirect link with FGFR inhibition improving MCT and ASL depth. We have shown previously that TGF-β signaling, which is implicated in aging processes in other lung diseases (54, 55), leads to a decrease in ASL volume, and pirfenidone, a therapy for IPF, can restore ASL volume (56). In this manuscript, we show that senolytic therapy with D+Q can improve MCT in an ex vivo CF rat model to further support a potential relation between senescence and mucociliary dysfunction (Figure 10). It is not clear how exactly D+Q achieves the effect in our model system; we did not see changes in cell number in vitro. Further studies are needed to define whether this effect could be mediated via MAPK p38. Cell senescence is one hallmark of aging, and several other hallmarks will be worth investigating in future studies, including other potential aging-related contributing pathological mechanisms such as the length-associated transcriptome imbalance (57). There are several additional limitations of our study. The most marked differences were seen in CF donors harboring the ΔF508 mutation. The hg551D rat model did not replicate those findings, but the *Cftr^–/–^* rat airways exhibited an increase in our studied cell senescence markers. Both ex vivo and in vitro models showed attenuation with FGFR inhibition, pointing to the role of FGFRs but not excluding additional contributing pathways, such as inflammation itself or ER stress. Furthermore, mainly basal and secretory cells exhibited the senescent state, and our studies assessing mucociliary function were done ex vivo and not in the primary cell cultures. Regulation of MCT is complex, and with secretory cells not showing marked senescence features, we assume that senescence of secretory cells could contribute to the decrease in ASL volume and unfavorable mucus composition impeding CBF.

Therefore, FGFRs could potentially represent a novel target for senolytic therapies, which are applicable for all pwCF independent of their mutations and may become increasingly relevant in a time of lengthening patient lifespan.

## Methods

### Animals

#### Sex as a biological variable.

Our study examined male and female animals, and similar findings are reported for both sexes. Both cell donor sexes were included for analyses.

#### Models.

All experiments used male and female Sprague-Dawley *Cftr*^tm1sage^ rats (*Cftr^–/–^*) rats or wild-type littermate controls at 6 months old as previously described (33). For experiments involving treatment of *Cftr^–/–^* rats with AZD4547 (Selleck Chemicals), we divided *Cftr*^–/–^ rats into 2 groups (*n* = 8 each), treated with 12.5 mg/kg body weight AZD4547 dissolved in DMSO with 1% sodium carboxymethyl cellulose (Selleck Chemicals) or vehicle once daily for 5 days. Method of delivery was oral gavage, which has been used previously (58). hG551D rats (Envigo) were treated with ivacaftor (VX-770) (Selleck Chemicals) for 14 days at 30 mg/kg/d or 3% methylcellulose vehicle by oral gavage (37).

#### Ex vivo Cftr^–/–^ rat trachea culture.

Tracheae were isolated from 6-month-old *Cftr^–/–^* rats and carefully explanted to culture cassettes according to methods previously published (34). Those cultures were incubated for 5 days with PneumaCult media (STEMCELL Technologies) and then treated with either BLU9931 (0.1 μM) or a combination of dasatinib and quercetin at 100 nM and 2 μM for 24 hours, respectively. After treatment, the tracheae were imaged via µOCT, and the epithelial cell layer was removed and used for RNA and protein isolation.

### Inhibitors

The following inhibitors were used for in vitro, in vivo, and ex vivo experiments: AZD4547 (Selleck Chemicals), PD173074 (Selleck Chemicals), BLU9931 (Selleck Chemicals), SB203580 hydrochloride (Tocris Bioscience, Bio-Techne) (a selective inhibitor for p38 MAPK) (59), and dasatinib and quercetin (Selleck Chemicals).

### Cell culture

Both primary human bronchial epithelial cells and CFBEΔ508 (CFBEs) (60) were used for experiments and cultured on Snap well filters (Corning) or plates in medium consisting of minimum essential media with l-glutamine and Phenol red, supplemented with 10% heat-inactivated fetal bovine serum (Atlas Biologicals), 1% l-glutamine, 1% penicillin/streptomycin, and 0.2% plasmocin. Human bronchial epithelial cells from CF (ΔF508) and non-CF donors were provided by the Cell Culture Core of the UAB Cystic Fibrosis Research Center and cultured and differentiated on the ALI as previously described (13). Briefly, passages 1–2 of the primary cells were seeded, kept in submerged cultures for about 1 week to expand to the necessary cell numbers, split on filters, and differentiated at the ALI for 4–6 weeks until ciliated cells were observed and mucus was produced, in addition to assessment of transepithelial electrical resistance (13, 14, 56). In addition, we assessed proportions of cell types, including ciliated, basal, and secretory cells, at the time cultures were used for experiments (Supplemental Figure 2).

### HEMT treatment

CF primary human bronchial epithelial cells were cultured at the ALI and treated with tezacaftor (VX-661), elexacaftor (VX-445), and ivacaftor (VX-770) (Selleck Chemicals) for 72 hours at 3 μM, 1 μM, and 3 μM, respectively. The media with ETI were refreshed every 24 hours.

### Western blot

Protein lysates were collected using 1× RIPA buffer with 1× Halt protease and phosphatase inhibitor (Thermo Fisher Scientific). Proteins were separated on 4%–20% precast Ready Gels (Bio-Rad) and transferred onto PVDF membranes (Pierce, Thermo Fisher Scientific). Membranes were blocked with either 5% BSA or 5% low-fat milk depending on antibody manufacturer recommendations for 30 minutes, then incubated overnight with the following primary antibodies: rabbit anti-p21 (catalog 29478), rabbit anti–BCL-xL (catalog 2764S), rabbit anti-FGFR4 (catalog 8562S), rabbit total and phospho–anti-ERK1/2 (catalog 4695S and 9101S), rabbit total and phospho–anti-p38 MAPK (catalog 9212S and 4511S), rabbit total and phospho–anti-PLCγ1 (catalog 2822S and 8713S) (Cell Signaling Technology), mouse anti–β-actin–peroxidase (MilliporeSigma A1978), and rabbit anti-p16 (Proteintech 10883-1-AP) diluted according to the manufacturer’s recommendations. After 3 washes with TBS with Tween (TBST), membranes were incubated with goat anti-rabbit peroxidase-conjugated antibody (Invitrogen, Thermo Fisher Scientific) at 1:6,000 in either 5% low-fat milk or 5% BSA depending on primary antibody manufacturer recommendations for 1 hour. After 3 washes in TBST, the membranes were imaged by chemiluminescence on a ChemiDoc XRS system (Bio-Rad) and acquired using Image Lab software (Bio-Rad). ImageJ was used to measure densitometry of positive signals on the membranes.

### RNA extraction and quantitative real-time PCR

Total RNA was extracted from rat lungs, primary human cells, and CFBEs as previously described (22, 27). Real-time quantitative PCR was performed with the following TaqMan probes: IL-6 Hs00174131, IL-1β Hs01555410, CXCL8 Hs00174103, BCL2L1 Hs00236329_m1 GAPDH (4333764F), FGFR1 Rn01478647, FGFR2 Rn01269940, FGFR3 Rn00584799, FGFR4 Rn01441815, BCL2 Rn99999125, p21 Rn00589996, p16 Rn00580664, IL-8 Rn00586403, IL-1β Rn00580432, IL-6 Rn01410330, BCL2L1 Rn06267811_g1, GAPDH Rn01775763 (Invitrogen, Thermo Fisher Scientific).

### Immunohistochemistry

Lungs from control and *Cftr^–/–^* rats were collected and fixed in 10% neutral buffered formalin for 24 hours followed by dehydration in ethanol for 24 hours. The tissue was then embedded in paraffin, cut into sections of 3–5 mm, and mounted on slides. Lung tissue slides were deparaffinized; stained using rabbit anti-p16, rabbit anti-p21 (Proteintech), anti-rabbit FGFR4 antibody (Santa Cruz Biotechnology, sc-136988), and rabbit anti-BCL-xL (Cell Signaling Technology); and developed using a rabbit-specific HRP/DAB detection IHC kit (Abcam), then counterstained with hematoxylin. The lung sections were stained with AB-PAS and hematoxylin and eosin by UAB Comparative Pathology core.

### Immunofluorescence

#### Epithelial subtype ratios and ALI colocalization.

Primary CF and non-CF bronchial epithelial cells were obtained from the UAB Cell Model and Evaluation Core upon ALI differentiation. The media were aspirated and washed with PBS prior to fixation with 4% paraformaldehyde. Staining methods were adapted from the Cell Signaling Technologies Immunostaining Protocol. Briefly, after fixation for at least 24 hours, cells were washed with PBS and permeabilized with 0.1% Triton for 15 minutes. The specimen was then washed and blocked with 1% BSA for 60 minutes prior to primary incubation overnight with the following antibodies: Krt5 (1:50, Invitrogen, Thermo Fisher Scientific, MA5-15348), uteroglobin (1:50, R&D Systems, Bio-Techne, MAB4218), FoxJ1 (1:50, Invitrogen, Thermo Fisher Scientific, PA5-36210 and 14-9965-82), p16 (1:100, Proteintech 10883-1-AP), and p21 (1:100, Cell Signaling Technology 29478). Secondary antibodies were added at 1:2,000 (anti-mouse, anti-rat, anti-rabbit) for 2 hours in the dark. Cells were then stained with NucBlue (Invitrogen, Thermo Fisher Scientific, R37606) for 5 minutes, concurrently mounted using Prolong Gold Mounting Media (Invitrogen, Thermo Fisher Scientific, P36930), and sealed with coverslips. After mounting, microscope slides were protected from light and stored at 4°C until imaging. Images were obtained on a Nikon Eclipse Ts2 with red, green, and blue cubes at original magnification, ×20, and cells were counted and subsequently analyzed using ImageJ and Excel, respectively.

Primary CF bronchial epithelial cells colabeled with a cell type marker and a senescence marker were imaged using a ZEISS Axio Observer with fluorescent monochrome Hamamatsu ORCA-Flash 4.0 LT camera and software Zen Blue. Images were obtained at ×40 original magnification with fixed exposure, gain, and signal threshold settings for each target. Signal threshold was based on negative controls stained for only the corresponding secondary antibodies. ImageJ 1.54d (61) and Fiji (62) were used for image processing.

#### Rat lung tissue staining.

Rat lung tissue sections were colabeled with a cell type marker and a senescence marker and were imaged using a ZEISS Axio Observer with fluorescent monochrome Hamamatsu ORCA-Flash 4.0 LT camera and software Zen Blue. Images were obtained at ×40 original magnification with fixed exposure, gain, and signal threshold settings for each target. Signal threshold was based on negative controls stained for only the corresponding secondary antibodies. ImageJ 1.54d (61) and Fiji (62) were used for image processing.

#### Human lung tissue staining.

Formalin-fixed, paraffin-embedded (FFPE) CF and non-CF human lung tissue sections were obtained from the UAB Tissue Biorepository. The immunofluorescence protocol was adapted from a prior immunohistochemistry staining protocol (63) and the Abcam Immunofluorescence Protocol. Briefly, FFPE sections were melted for 50 minutes in an oven set for 60°C. Slides were then deparaffinized and rehydrated using Clear-Rite 3 (Epredid, 6901) and 100%, 95%, and 70% ethanol. Antigen retrieval was obtained using antigen unmasking solution (100×, citrate; Vector Laboratories H3300) in a steamer for 20 minutes. Tissue sections were then rinsed once in PBS and permeabilized for 5 minutes using 0.1% Triton in PBS prior to blocking in 5% BSA. Tissues were then incubated overnight in primary antibodies Krt5 (1:50, Cytokeratin 5, Invitrogen, Thermo Fisher Scientific), uteroglobin (1:50, anti-human uteroglobin, R&D Systems, Bio-Techne), or FoxJ1 (1:50, Anti-Hu/Mo FOXJ1, Invitrogen, Thermo Fisher Scientific, 14-9965-82) and rabbit p16 (1:200, Proteintech) as described above. After seven 5-minute 1× PBS washes, the tissue sections were incubated for 45 minutes with the respective secondary antibodies at 1:2,000: alpaca anti-Mouse IgG1 (VHH), Alexa Flour 647 - Invitrogen, Thermo Fisher Scientific (SA5-10333); goat anti-rat IgG (H+L), Alexa Flour 488 - Invitrogen, Thermo Fisher Scientific (A-11006); goat anti-rabbit IgG (H+L), Alexa Flour 568 - Invitrogen, Thermo Fisher Scientific (A-11011). Seven 5-minute 1× PBS washes were completed again, and then tissue sections were incubated with diluted NucBlue (2 drops in 1 mL PBS, Invitrogen, Thermo Fisher Scientific) for 10 minutes before mounting with Prolong Gold Mounting Media and sealing with coverslips. After mounting, microscope slides were protected from light and stored at 4°C until imaging. Imaging was done on a Nikon Eclipse Ts2 at either ×10 or ×40 original magnification to assess colocalization.

### ELISA

ELISAs for the quantitative recognition for IL-6 and IL-8 (Invitrogen, Thermo Fisher Scientific) were performed using supernatant from CFBEs after treatment with the different inhibitors as outlined before. ELISA for IL-8 (Abcam) was performed on protein lysates normalized to 2 mg/mL of total protein in each sample from sham- and AZD4547-treated *Cftr^–/–^* rats.

### scRNA-Seq data

Publicly available scRNA-Seq data set GSE150674 that was previously aligned, filtered, normalized, and annotated was used to analyze senescence scores using 3 separate gene sets (18). We utilized the total data set of 19 controls and 19 CF donors with end-stage lung disease undergoing lung transplantation. For the analysis, we separated the CF group by CF cells that are ΔF508 homozygous (*n* = 10,131 cells and *n* = 8 donors) with a 34- to 36-year-old average age for the CF group and compared this CF ΔF508 homozygous group with the healthy donor group (*n* = 23,119 cells and *n* = 19) with an average age of 46 years for the control group. Both groups include male and female donors. Using BBrowser 3 software (Bio Turning) and 3 different sets of gene list associated with cellular senescence, we created senescence scores visualized via UMAP and violin plots. The gene sets used in the senescence scores were CellAge senescence genes database, which was filtered for genes that induce cellular senescence (19); SenMayo (20); and cellular senescence markers used in this study: *CDKN1A*, *CDKN2A*, *BCL2*, *BCL2L1*, *IL6*, *IL1B*, and *GLB1*. Violin plots were made by extracting the signature score data from BBrowser3 and analyzed in Prism 9 (GraphPad).

### SA-β-gal staining

Cytochemical staining for SA-β-gal was performed using a Senescence associated β-galactosidase Staining kit (Cell Signaling Technologies 9860) following the protocol provided. Rat tissue slides were counterstained with DAPI (Vector Laboratories). To quantify the amount of β-galactosidase staining, we captured 3 images from different regions of the cell culture plates and counted all cells and all β-galactosidase–positive cells from the 3 images and made a ratio of β-galactosidase–positive cells to total cells counted. CFBE experiments were done in triplicates, and primary cell cultures included 3 different donors from CF and non-CF.

### μOCT

Measurements of functional microanatomic parameters in CF primary human bronchial epithelial cells on ALI and ex vivo tracheae were performed using μOCT, a high-resolution microscopic reflectance imaging system as previously described (64).

### Statistics

Data were analyzed with Prism 9 (GraphPad) as previously described (27) using unpaired 2-tailed Student’s *t* test or 1-way ANOVA for a minimum 3 independent experiments in duplicate. Data are shown with individual values from each experiment ± SEM. Statistical significance was accepted at *P* value of less than 0.05.

### Study approval

The Institutional Animal Care and Use Committee at the UAB approved all animal protocols. For cells, the UAB waived the need for written consent.

### Data availability

All data associated with this manuscript are present in the paper and Supporting Data Values file. The scRNA-Seq data set is publicly available (accession GSE150674) and cited in this manuscript.

## Author contributions

ME, SK, MJH, and JWB contributed to the conceptualization or design of the study. ME, EH, MJH, SB, SEB, SV, ELM, LIJ, and PHH contributed to the acquisition of the data, and ME, MJH, EH, LIJ, PHH, YYS, DJT, JWB, and SK contributed to the analysis and interpretation. ME, MJH, JWB, and SK drafted the manuscript. All authors contributed to the article and approved the submitted version.

## Supplementary Material

Supplemental data

Unedited blot and gel images

Supporting data values

## Version 1 06/25/2024

In-Press Preview

## Version 2 08/08/2024

Electronic publication
