# Supplementary material for: FGF receptors mediate cellular senescence in the cystic fibrosis airway epithelium
Source: JCI Insight. 2024 Jun 25;9(15):e174888. doi: 10.1172/jci.insight.174888 (PMC11383597; doi:10.1172/jci.insight.174888)
Supplement: Supplemental data [file jciinsight-9-174888-s042.pdf]

A

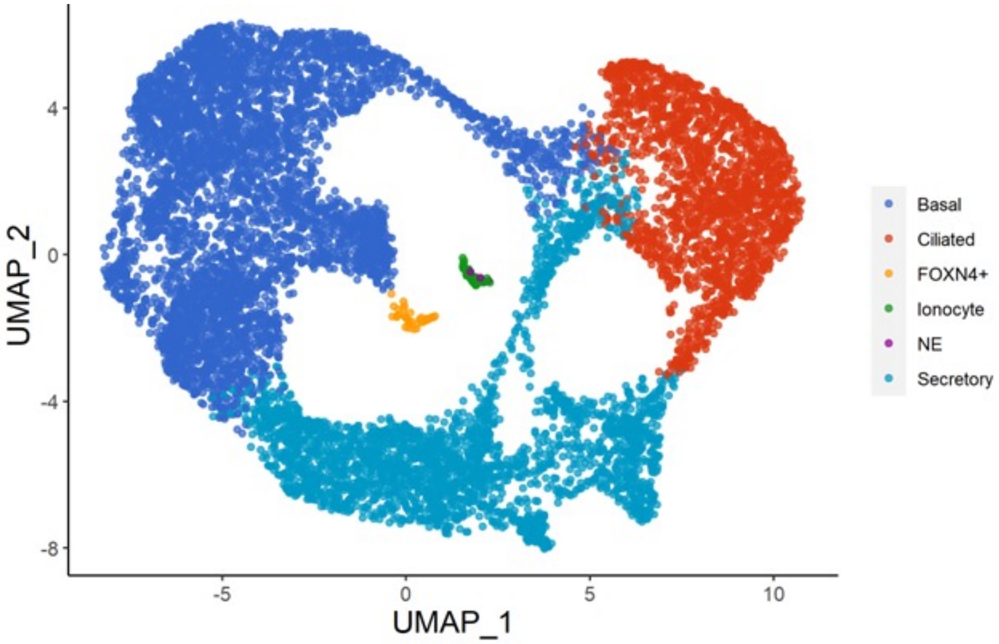

B

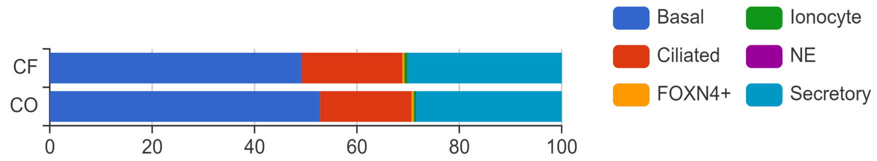

Supplemental Figure 1: Single-cell RNA sequencing data showing the abundance of different epithelial subtypes comparing CF and non-CF cells. (A) scRNA UMAP from GSE150674 labelling the subset populations of epithelial cells. (B) Distribution of different cell types from the dataset between control (CO) and CF donors (CF).

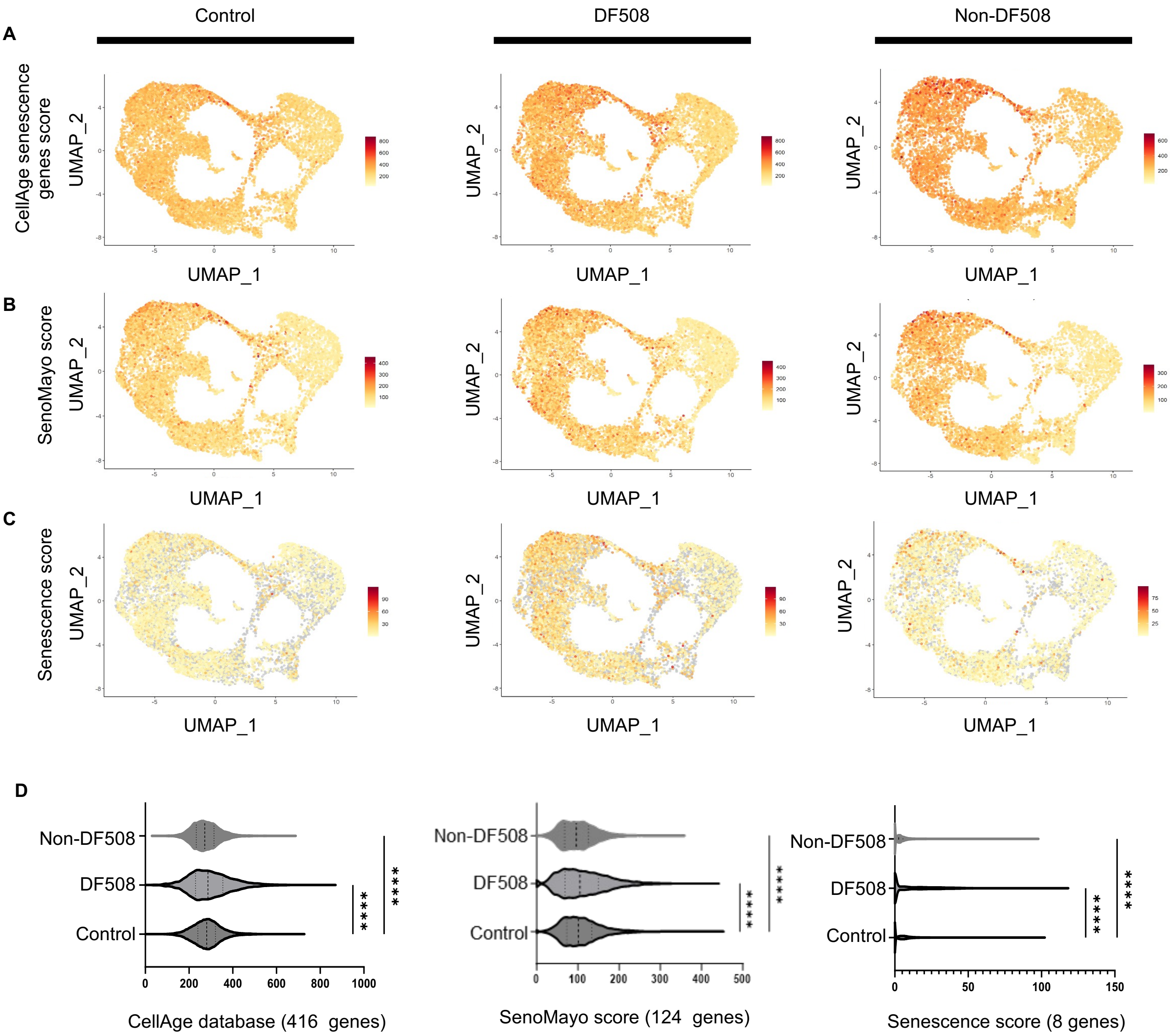

Supplemental Figure 2: Single-cell RNA sequencing data revealed an increase in cell senescence markers in CF airway cells homozygous for the DF508 mutation. Single cell RNA (scRNA) transcriptome of control and CF epithelial cells (GSE150674 Control (CO) and Cystic Fibrosis (CF) epithelial cell count CO n= 23119 and CF n= 17590) from 19 control and 19 CF donor lungs from donors with end stage CF lung disease and healthy controls, CF ΔF508 homozygous patients (n=10,131 cells and n=8 donors), healthy donors (n=23119 cells and n=19 donors) and Non- ΔF508 homozygous CF patients (n= 7,459 cells and n= 11 donors).were separated from the whole dataset and analysed using BBrowser3 to generate UMAPs and violin plots comparing control cells, ΔF508 cells and non- ΔF508 cells for senescence scores from three separate gene databases: (**A,D)**:CellAge database of senescence inducing genes (416 genes), **(B,D)** SenMayo (124 genes) and (**C,D):** cellular senescence markers used in this study (*CDKN1A, CDKN2A, BCL2, BCL2L1, IL6, IL1B and GLB1*). Statistical analysis was done using one-way ANOVA showing with *p < 0.05, **p < 0.01, ***p < 0.001 and **** p<0.0001.

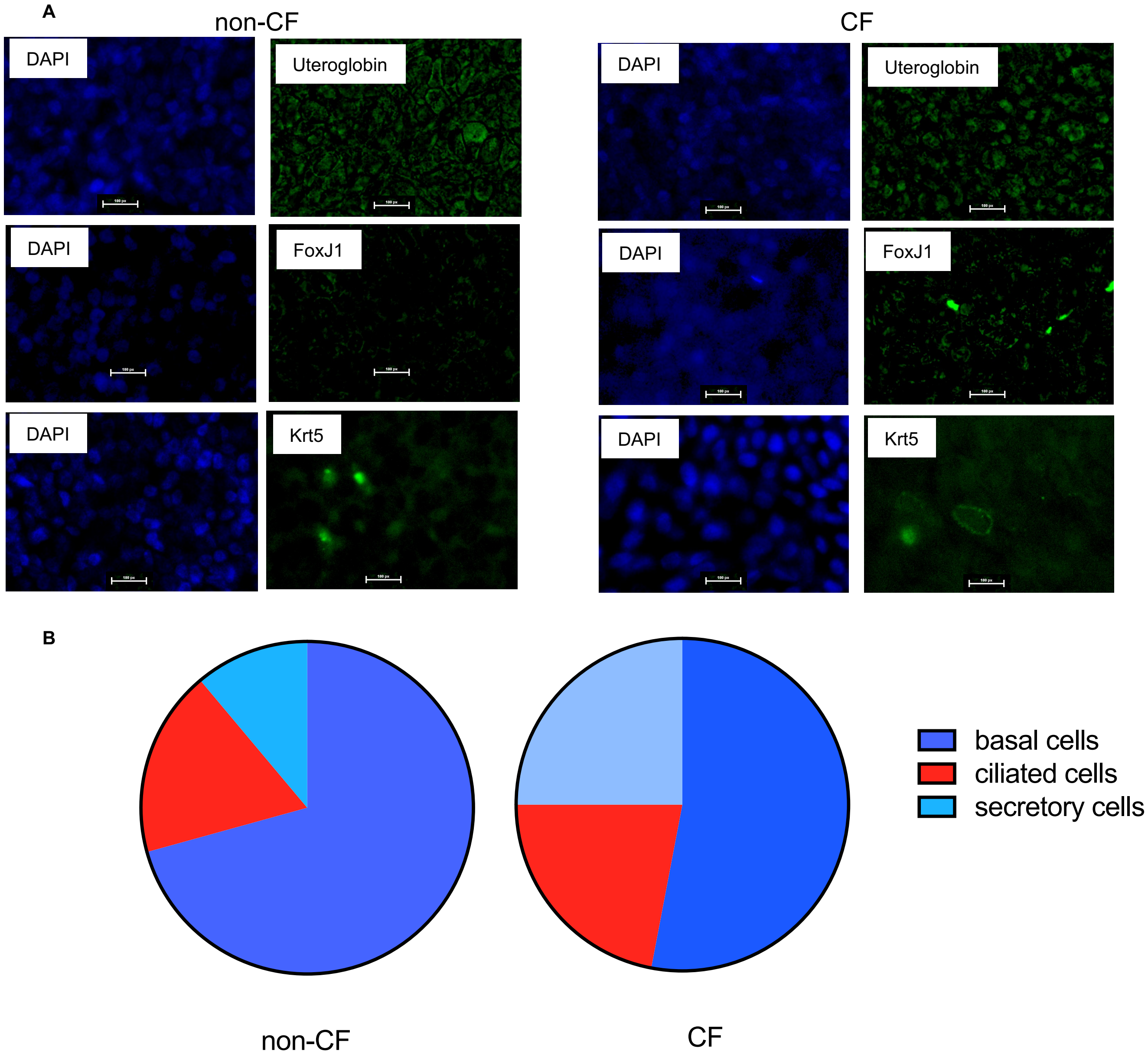

Supplemental Figure 3: Immunofluorescence staining of primary CF ALI cultures using markers for basal, ciliated and secretory cell subtypes. (A): Immunofluorescence staining of human primary non-CF and CF ALI cultures with DAPI to visualize cell nuclei, anti-uteroglobin, a marker for basal cells, anti-FoxJ1, a marker for ciliated cells, and anti-Krt5, a marker for secretory cells (B) Quantitative distribution of the three different cell types between control (non-CF = 3 donors) and CF donors (CF = 3 donors) .

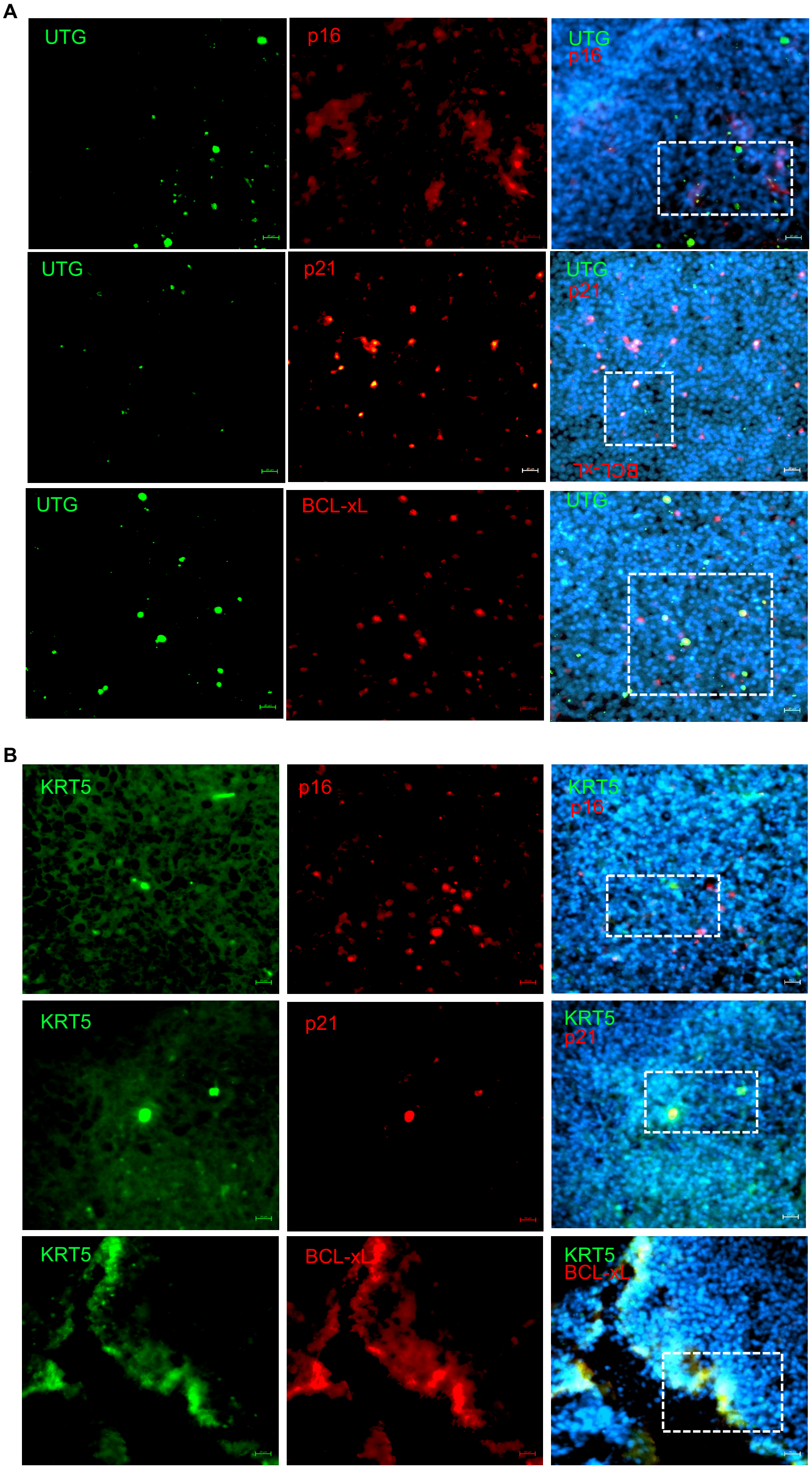

Supplemental Figure 4: Co-expression of senescence markers in basal and secretory cells in primary human CF ALI cultures. (A) Immunofluorescence staining for p16, p21 and BCL-xL and co-labelling with Uteroglobin (UTG; secretory cells) and (B) KRT5 (KRT5: basal cells) in primary human CF ALI cultures and nuclear staining with DAPI.

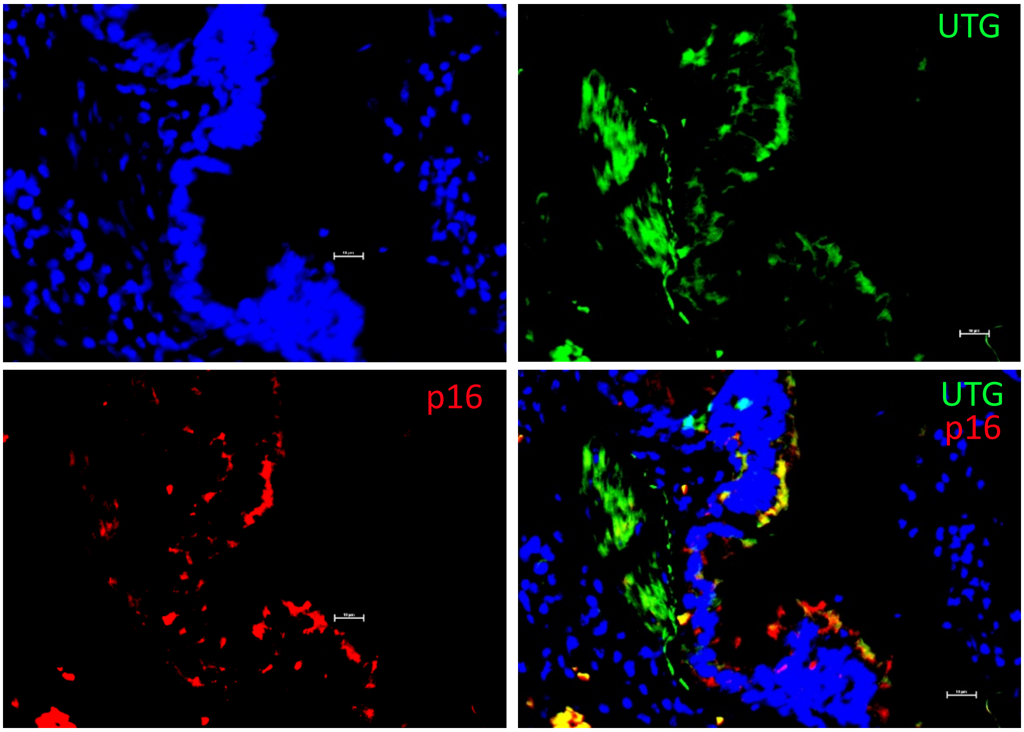

Supplemental Figure 5: Expression of p16 in secretory cells in human CF lung tissue. Immunofluorescence staining for p16 and co-labelling with Uteroglobin (UTG; secretory cells) in paraffin embedded human CF lung tissue and nuclear staining with DAPI.

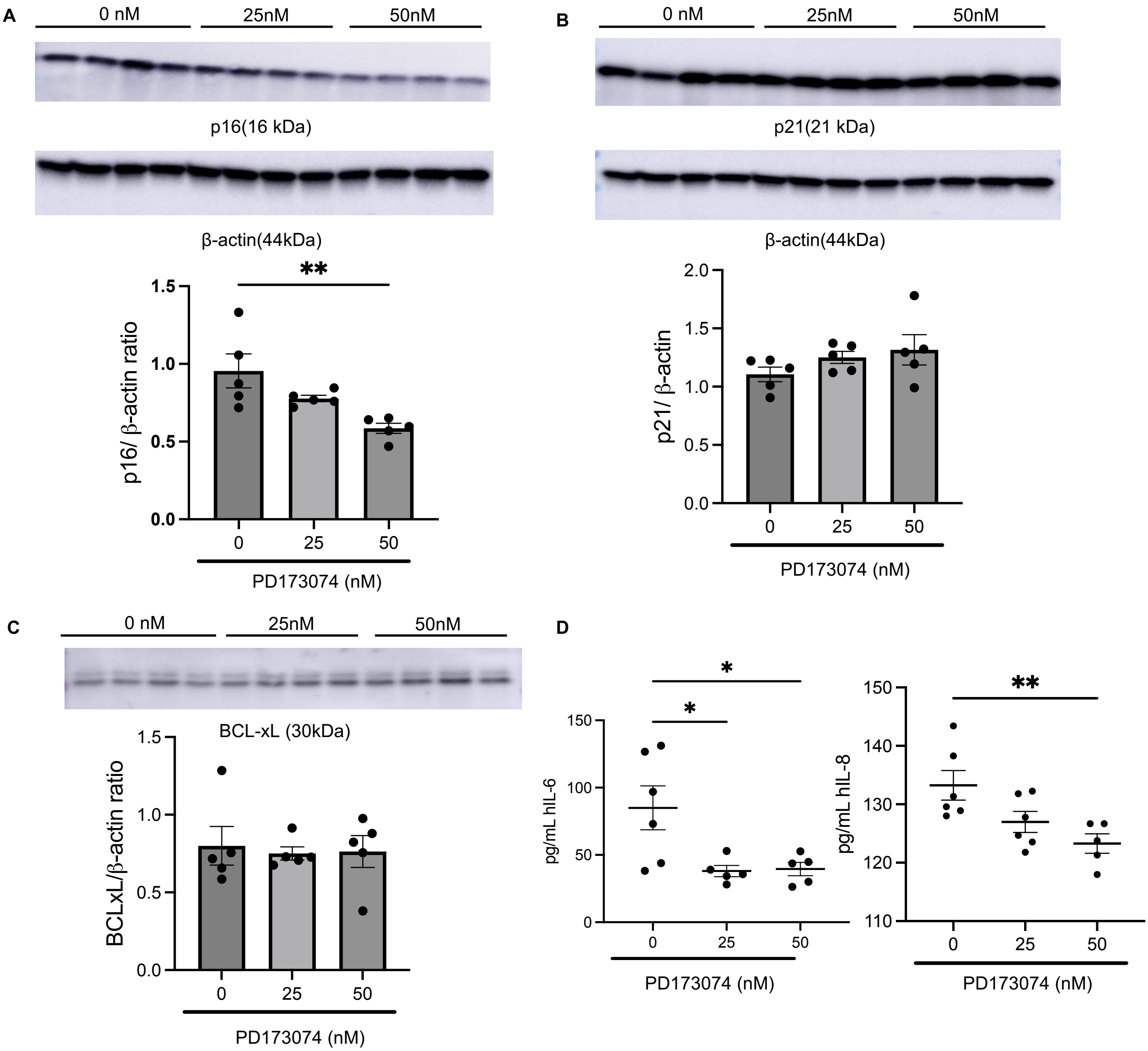

Supplemental Figure 6: PD173074 targeting FGFR1 inhibition downregulated p16, IL-6, and IL-8 in CFBEs. (A) Representative immunoblot images and bar graphs showing densitometric analyses for p16, (B) p21 and (C) BCL-xL from ALI cultures from CF ΔF508 and non-CF donors (the same β-actin loading control was used for B and C). (D) Dot plots showing protein levels of IL-6 and IL-8 from basolateral media of the same CF ΔF508 and non-CF donor ALI cultures after treatment with PD173074 for 24 hours (vehicle = 0, 25, and 50 nM). Statistical analysis was done using Student's t-test showing means ± SEM with *p < 0.05, **p < 0.01, and ***p < 0.001 from 5-6 different donors per group.

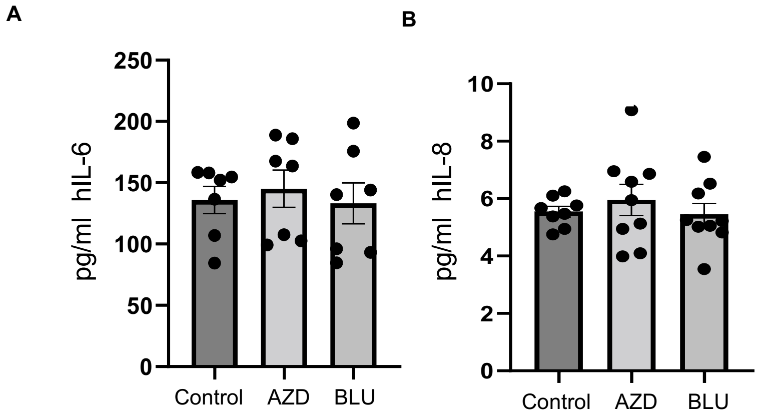

Figure 7: FGFR inhibition does not affect secreted IL-6 and IL-8 levels in CFBEs. (A) IL-6 and (B) IL-8 protein levels in supernatant from CFBEs, which were treated with AZD4547 0.1uM or BLU9931 0.1uM for 24 hours, which showed no significant difference between control and treated cells. Statistical analysis was done using Student's t-test showing means ± SEM with n = 7 independent experiments.

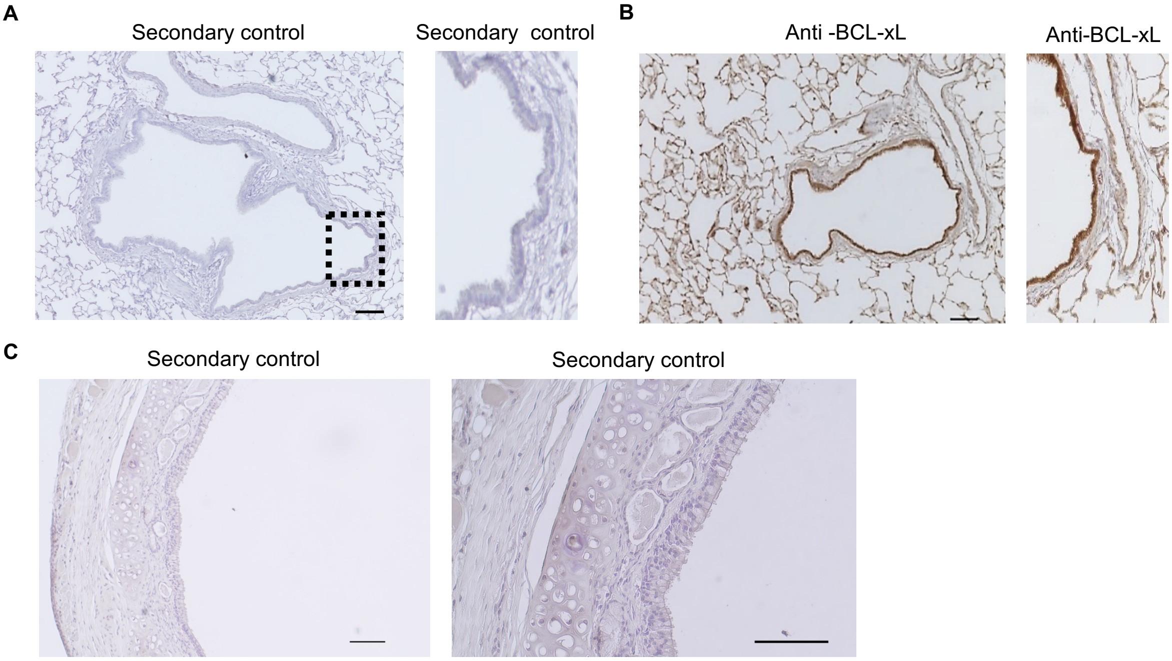

Supplemental Figure 8: Immunohistochemistry of *Cftr*$^{-/-}$ rat tissue and *Cftr*$^{-/-}$ rat trachea tissue with secondary controls only.

(A) Immunohistochemistry of *Cftr*$^{-/-}$ rat tissue with secondary only as control for Figure 5, shown here in comparison to (B) anti-BCL-xL staining in *Cftr*$^{-/-}$ rat tissue compared to A. (C) Immunohistochemistry of *Cftr*$^{-/-}$ rat trachea tissue with secondary only as control at 20X and 40X magnifications, scale bar is 100 μm.

A

Rat lung tissue - Secondary only negative controls (40x)

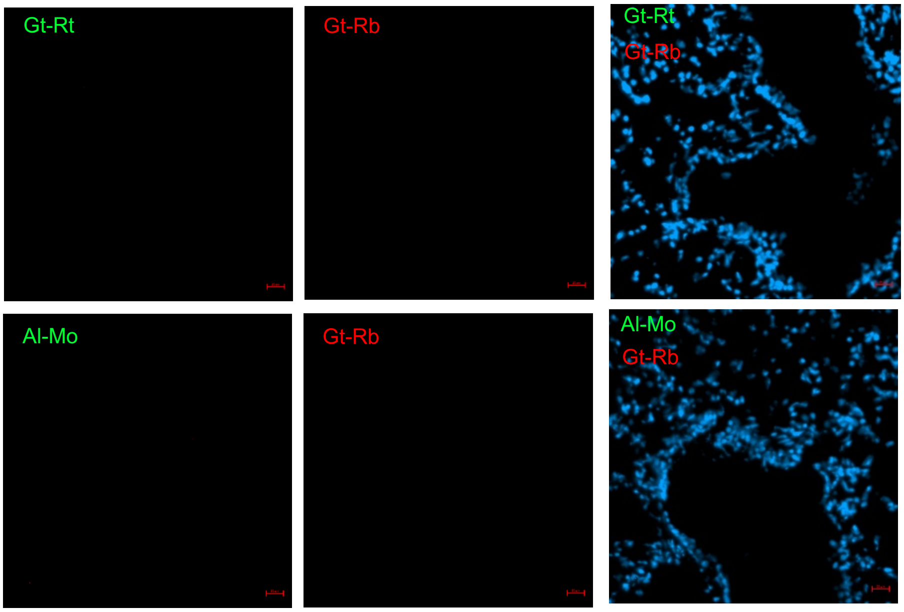

B

Primary CFBEs- Secondary only negative controls (40x)

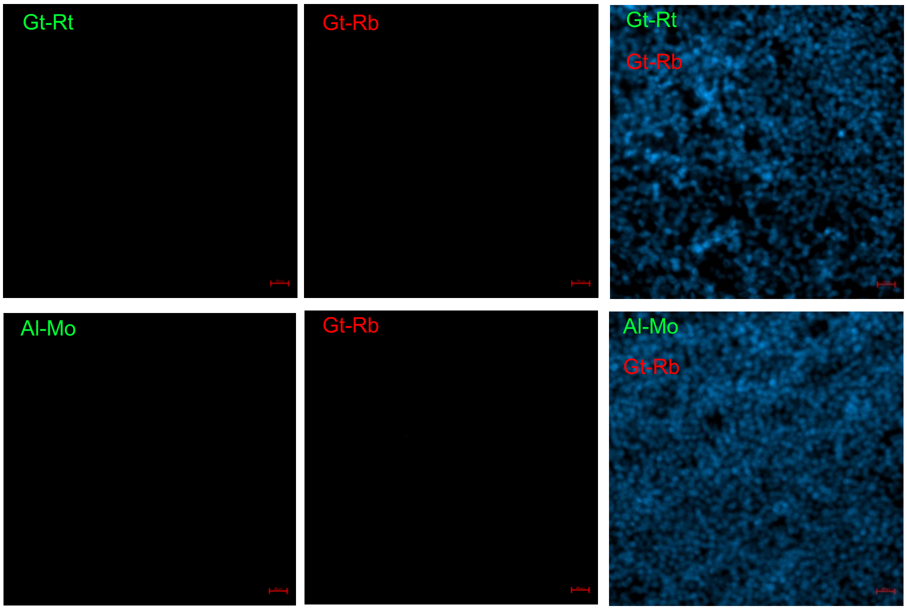

Supplemental Figure 9: Immunofluorescence staining of *Cftr*$^{-/-}$ rat tissue and primary ALI culture using secondary control antibodies only. (A) Immunofluorescence controls of *Cftr*$^{-/-}$ rat tissue and (B) primary ALI cultures using DAPI for nuclear stain, and secondary goat anti-rat (Gt-Rt), goat anti-rabbit (Gt-Rb) and alpaca anti-mouse (Al-Mo) antibodies at 40X magnifications.

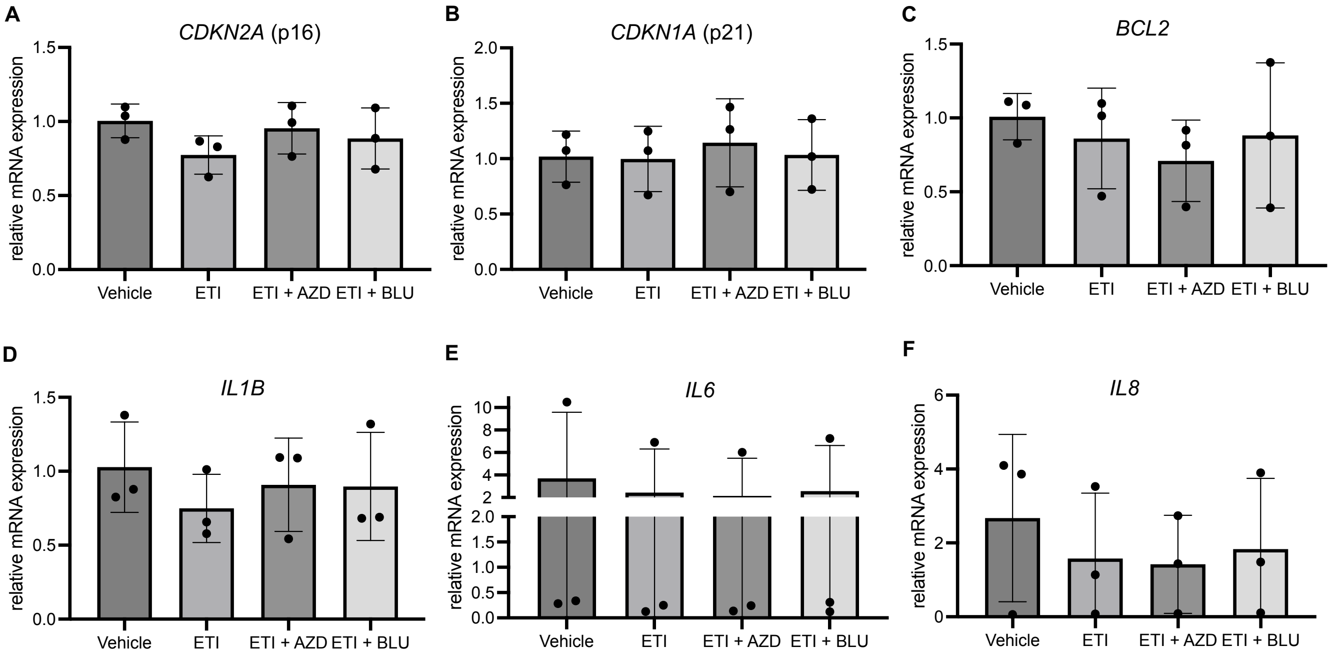

Supplemental Figure 10: Expression of senescence markers did not change in CFBEs with ETI treatment or with a combination of ETI+FGFR inhibitors. Relative mRNA levels of (A) *CDKN2a* (p16), (B) *CDKN1a* (p21), (C) *BCL2,* (D) *IL1B*, (E) *IL6*, and (F) *IL8* from CFBEs treated with ETI (VX-661/VX-445/VX-770) for 72 hours versus vehicle control or a combination of ETI with either AZD4547 (0.1 μM) or BLU9931 (0.1 μM). Statistical analysis was done using Student's t-test showing means ± SEM from 3 different experiments.

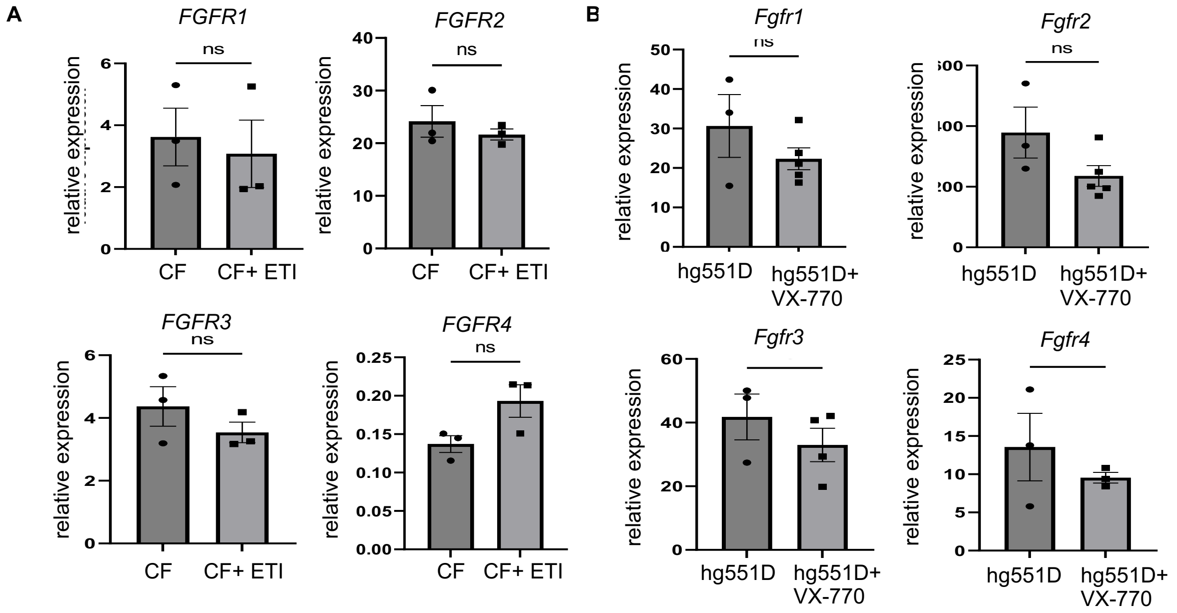

Supplemental Figure 11: ETI treatment did not affect FGFR mRNA levels *in vitro* or *in vivo.* (A) Relative mRNA levels of *FGFR1-4* in CF primary human bronchial epithelial cells treated with VX-661/VX-445/VX-770 (ETI) for 72 hours versus vehicle control. (B): Relative mRNA levels of *Fgfr1-4* from total lung tissue of hg551D rats treated with VX-770 and compared to vehicle treated hg551D rats. Statistical analysis was done using Student's t-test showing means ± SEM from 3 different donors per group and 3-5 different rats per group.

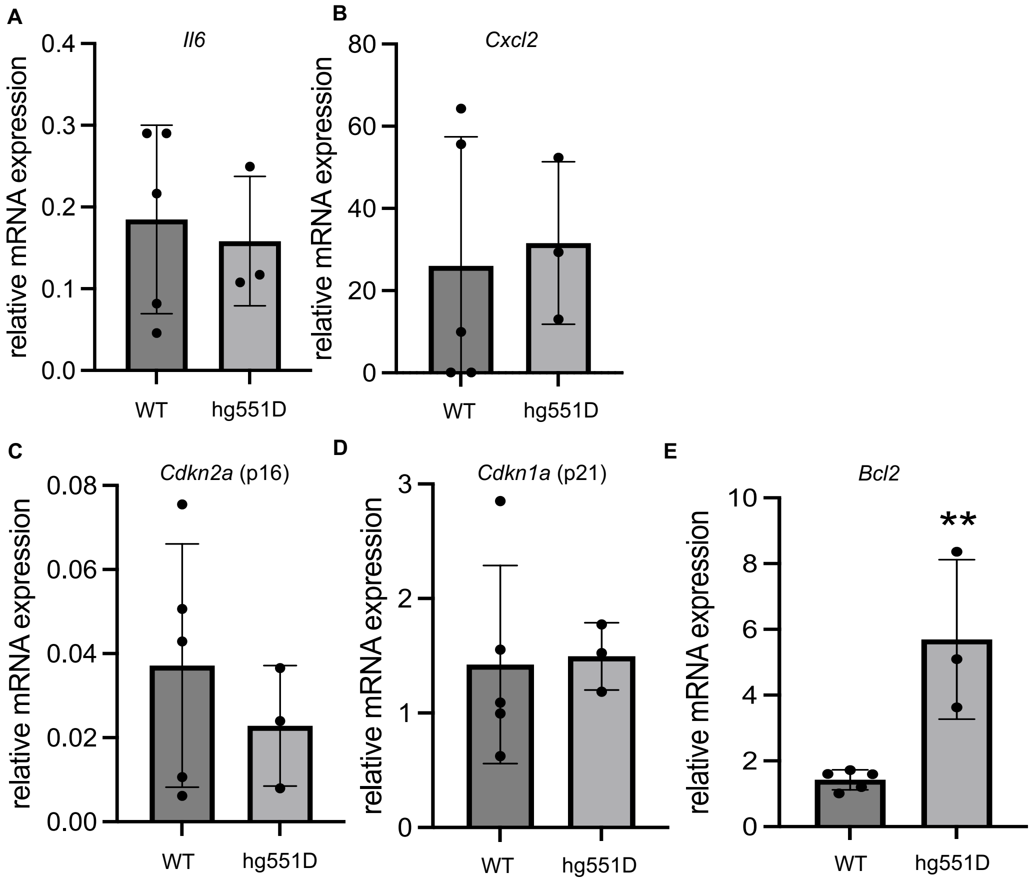

Supplemental Figure 12: hg551D rats do not exhibit elevated levels of cellular senescence compared to littermate controls.

Relative mRNA levels of (A) *Il6*, (B) *Cxcl2,* (C) *Cdkn2a* (p16), (D) *Cdkn1a* (p21), (E) Bcl2 from total lung tissue of control (WT) and hg551D rats. Statistical analysis was done using Student's t-test showing means ± SEM from 3-8 different rats per group.

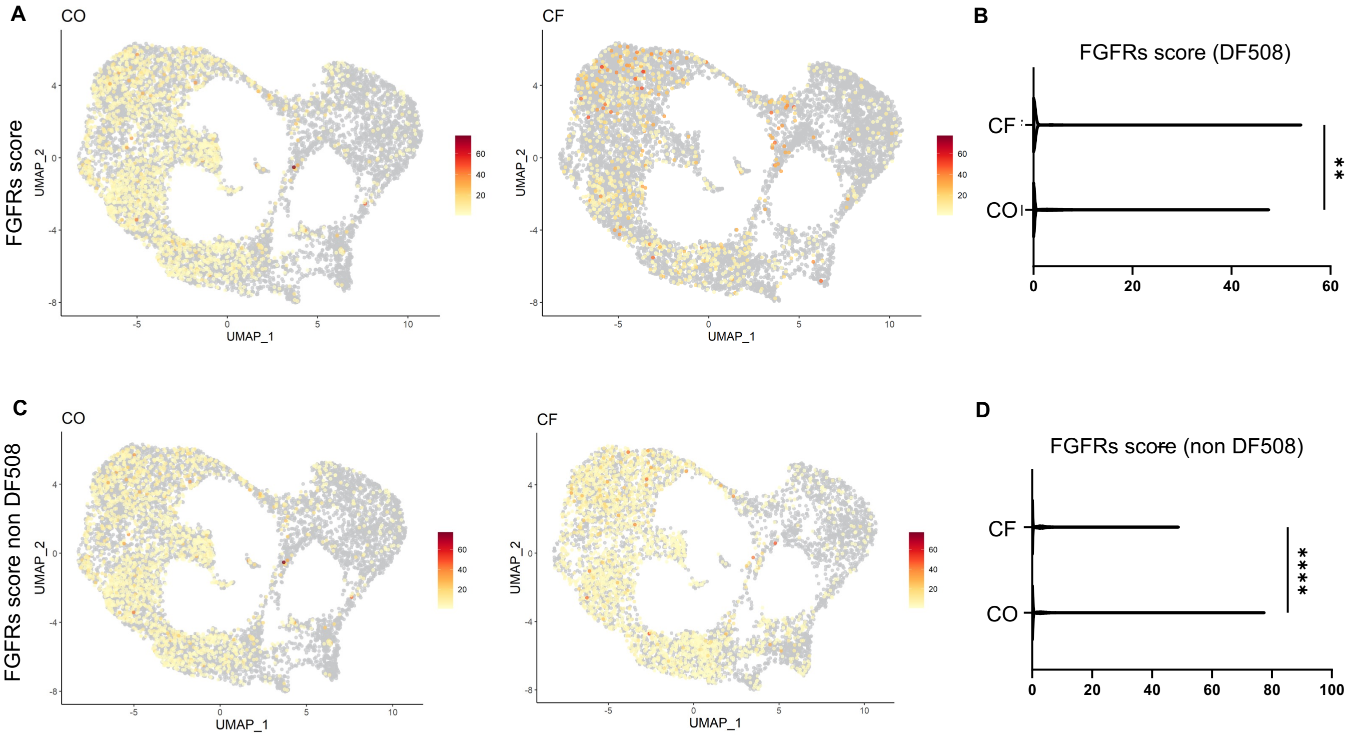

Supplemental Figure 13: Single-cell RNA sequencing data revealed an increase in FGF receptors in CF epithelial cells compared to control epithelial cells. UMAPs and violin plots showing FGFR 1, 2, 3 and 4 in (A,B) DF508 CF epithelial cells and (C,D) CF epithelial cells from donors with all other mutations in comparison to epithelial cells from control donors. Statistical analysis was done using Student's t-test or one-way anova showing with **p < 0.01, and **** p<0.0001.

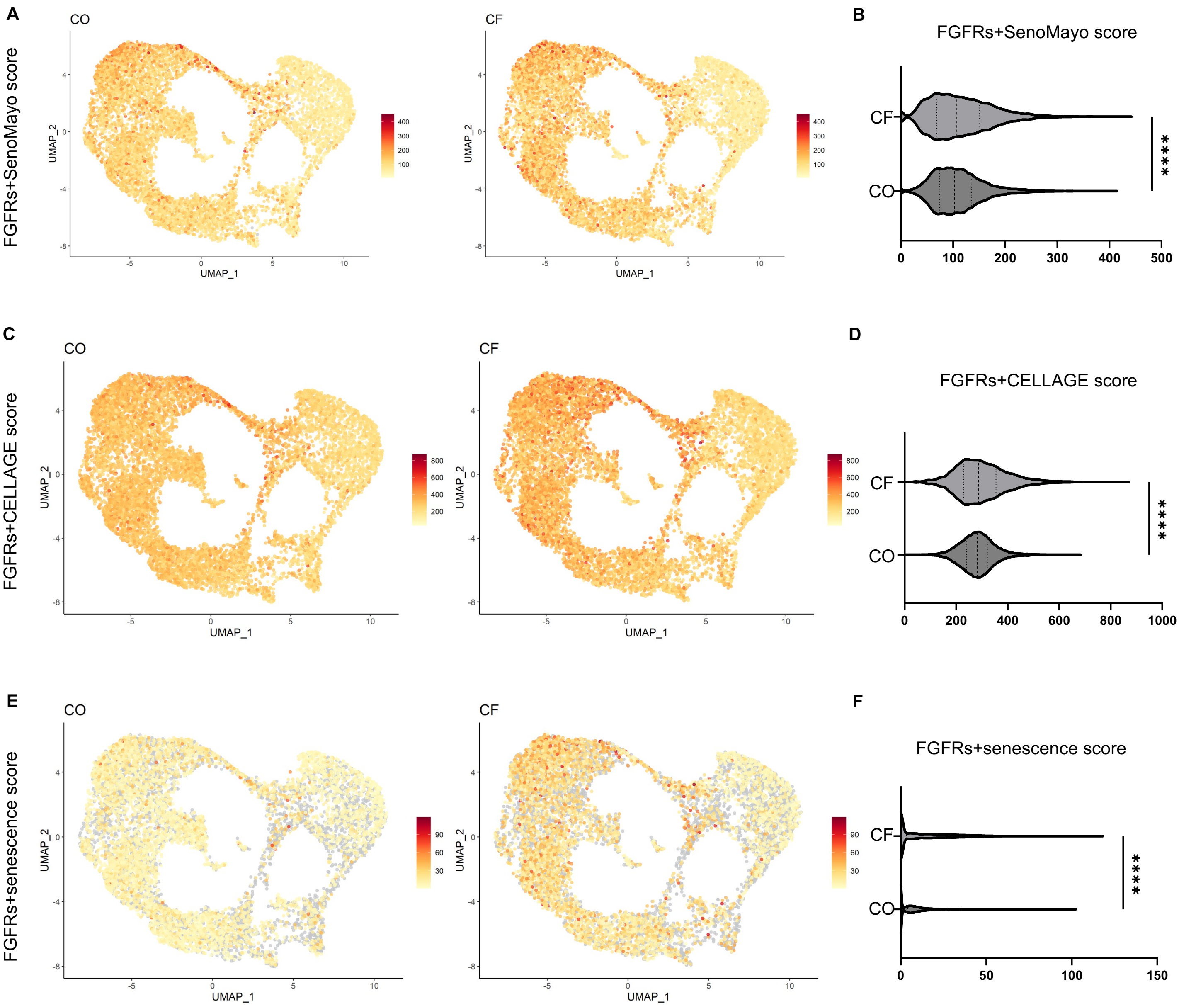

Supplemental Figure 14: Single-cell RNA sequencing data revealed an increase in cell senescence markers together with FGFRs in CF epithelial cells compared to control epithelial cells. UMAPs and violin plots showing senescence scores together with FGFRs from three separate gene databases: (A,B) CellAge database of senescence inducing genes, (C,D) SenoMayo and (E,F) cellular senescence markers used in this study (*CDKN1A, CDKN2A, BCL2, BCL2L1, IL6, IL1B and GLB1*). Statistical analysis was done using Student's t-test or one-way Anova showing with *p < 0.05, **p < 0.01, ***p < 0.001 and **** p<0.0001.

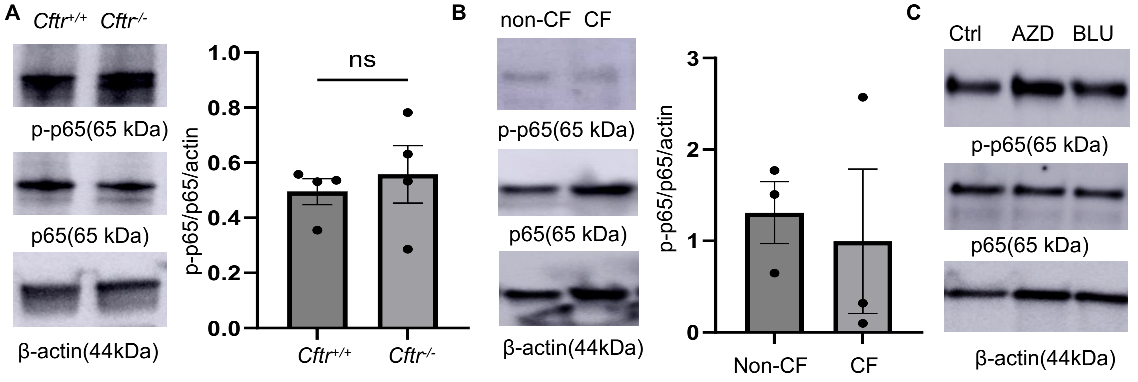

Supplemental Figure 15: Phosphorylated p65 was not affected by *Cftr* knockout/deficiency or FGFR inhibition. Representative immunoblot of phospho-p65, p65 and β-actin and densitometric analysis of (A) total lung tissue from *Cftr*$^{+/+}$ and *Cftr*$^{-/-}$ rats (n = 4), from (B) primary ALI cultures from control and CF donors (n = 3) and from (C) primary CF ALI cultures, treated with either AZD4547 or BLU9931 for 24h (n = 3). Statistical analysis was done using Student's t-test showing means ± SEM from 3 different donors per group and 3-5 different rats per group.
