# Supplementary material for: FGF receptors mediate cellular senescence in the cystic fibrosis airway epithelium
Source: JCI Insight. 2024 Jun 25;9(15):e174888. doi: 10.1172/jci.insight.174888 (PMC11383597; doi:10.1172/jci.insight.174888)
Supplement: Unedited blot and gel images [file jciinsight-9-174888-s043.pdf]

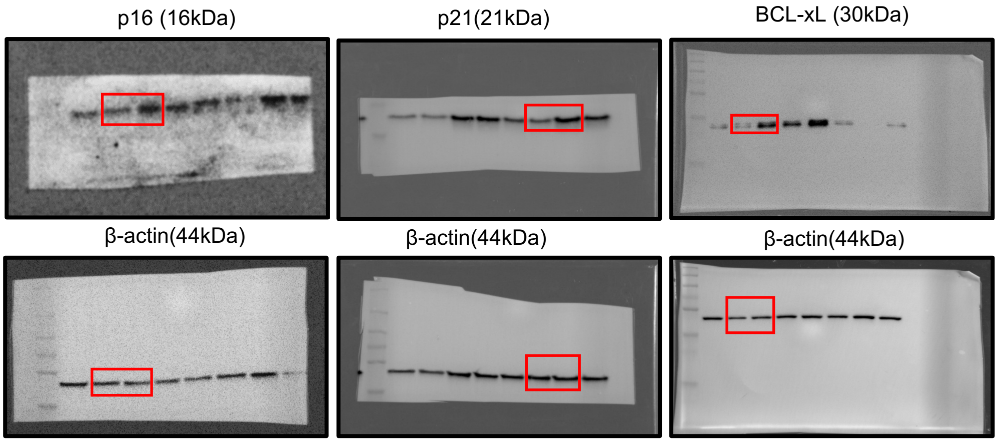


**Original western blot images for Figure 2A.** For the manuscript, a horizontal region was cropped for the bands of interest indicated by a red box on each picture. No additional modification was performed.

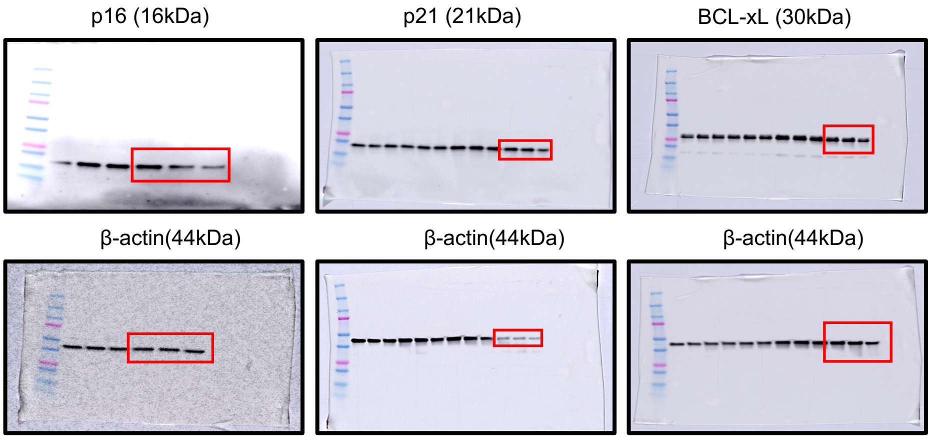


**Original western blot images for Figure 3A.** For the manuscript, a horizontal region was cropped for the bands of interest indicated by a red box on each picture. No additional modification was performed.

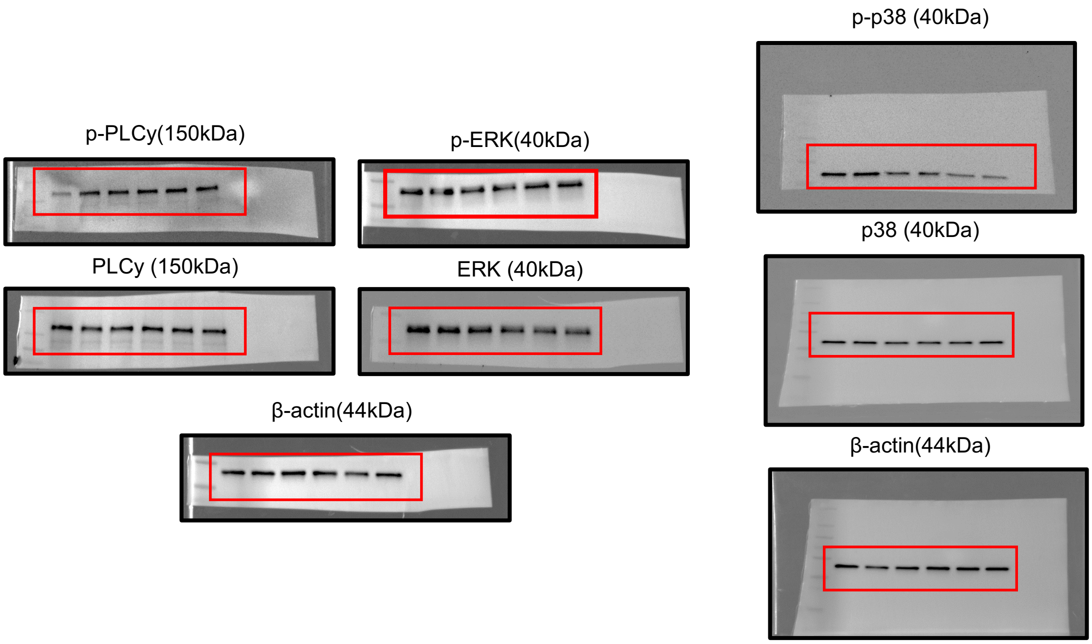


**Original western blot images for Figure 3C.** For the manuscript, a horizontal region was cropped for the bands of interest indicated by a red box on each picture. No additional modification was performed.

p-p38(40kDa) p38 (40kDa) β-actin(44kDa)

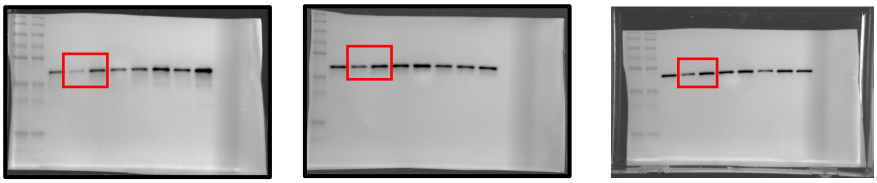

**Original western blot images for Figure 3D.** For the manuscript, a horizontal region was cropped for the bands of interest indicated by a red box on each picture. No additional modification was performed.

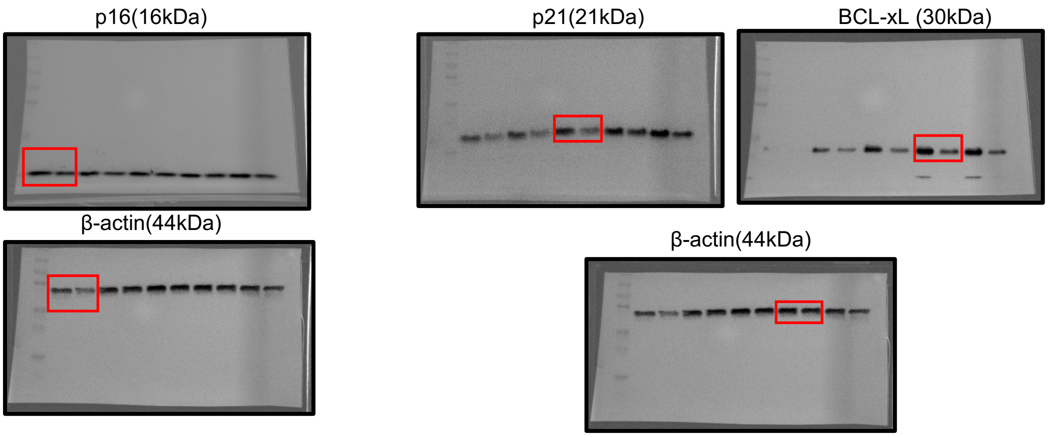


**Original western blot images for Figure 4A.** For these western blots, the same blots were used to probe for multiple of the proteins shown in the figure. For the manuscript, a horizontal region was cropped for the bands of interest indicated by a red box on each picture. No additional modification was performed.

FGFR4 (125 kDa)

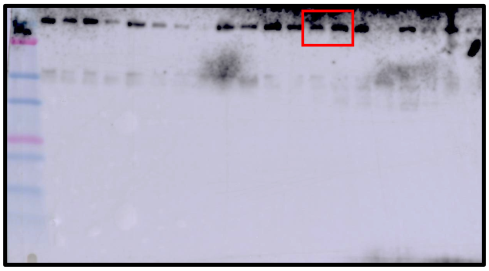

β-actin (42kDa)

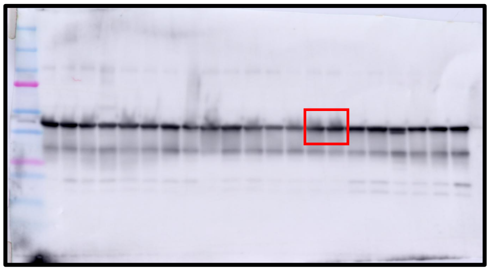

**Original western blot images for Figure 7C**. For the manuscript, a horizontal region was cropped for the bands of interest, indicated by a red box on each picture. No additional modification was performed.

p21(21kDa)

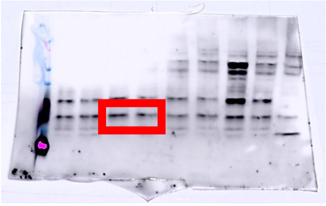

BCL-xL (30kDa)

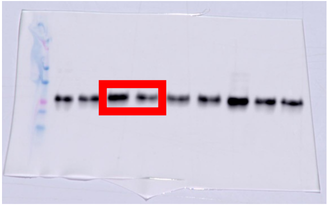

β-actin(44kDa)

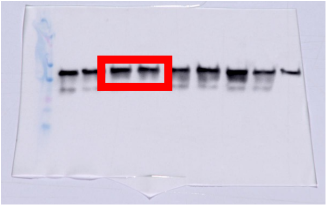

**Original western blot images for Figure 8B.** For these western blots, the same blots were used to probe for multiple of the proteins shown in the figure. For the manuscript, a horizontal region was cropped for the bands of interest, indicated by a red box on each picture. No additional modification was performed.

p-p38(40kDa)

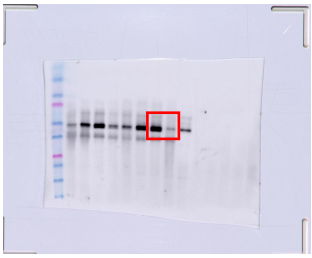

p38(40kDa)

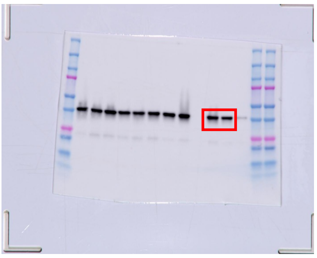

β-actin(44kDa)

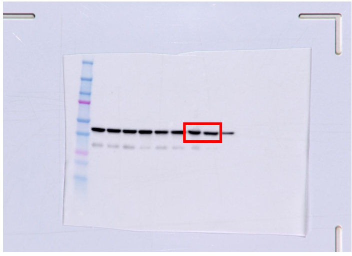

**Original western blot images for Figure 8C.** For the manuscript, a horizontal region was cropped for the bands of interest, indicated by a red box on each picture. No additional modification was performed.

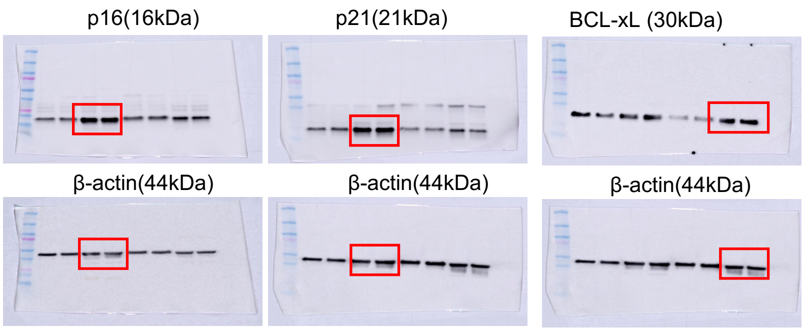


**Original western blot images for Figure 11B.** For these western blots, the same blots were used to probe for multiple of the proteins shown in the figure. For the manuscript, a horizontal region was cropped for the bands of interest, indicated by a red box on each picture. No additional modification was performed.

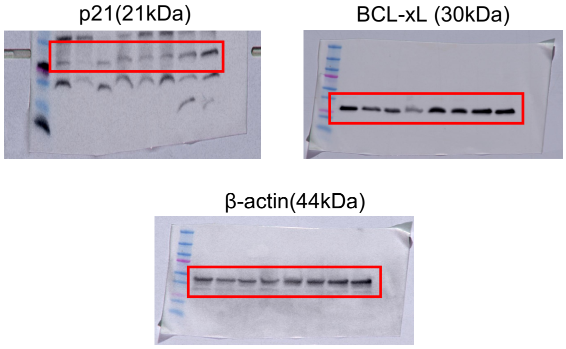


**Original western blot images for Figure 11D**. For these western blots, the same blots were used to probe for multiple of the proteins shown in the figure. For the manuscript, a horizontal region was cropped for the bands of interest, indicated by a red box on each picture. No additional modification was performed.

p16(16kDa)

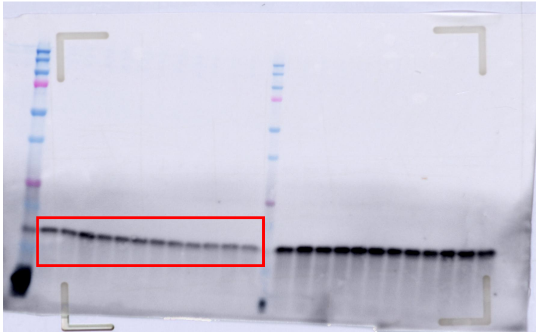

p21(21kDa)

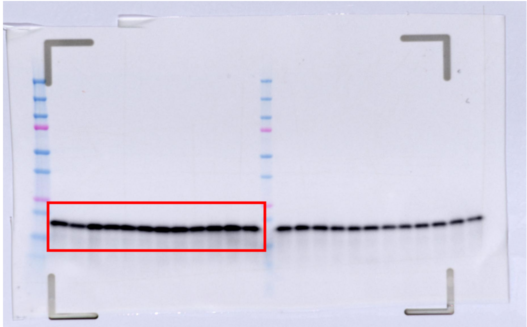

β-actin(44kDa)

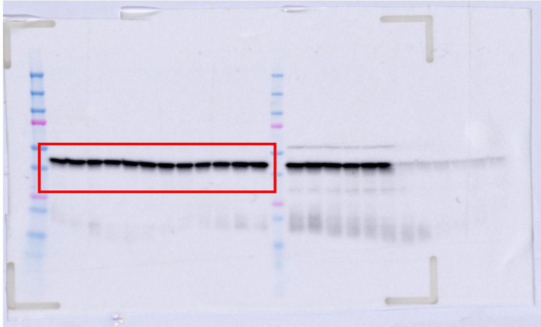

β-actin(44kDa)

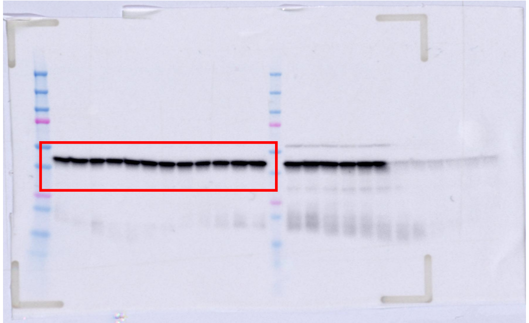

BCL-xL (30kDa)

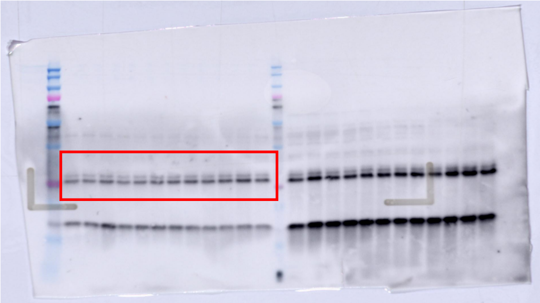

β-actin(44kDa)

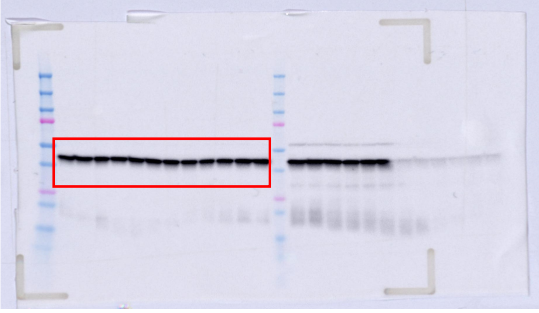

Blots for Supplemental Figure 6 A,B and C

# NFKB protein expression

P-p65(65Dka)

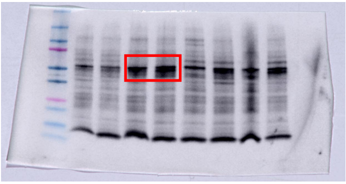

β-actin(44kDa)

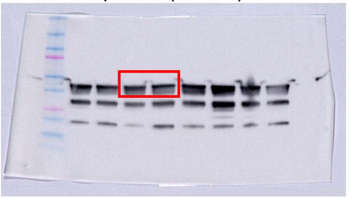

p65(65Dka)

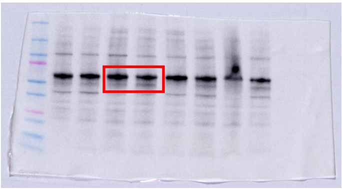

β-actin(44kDa)

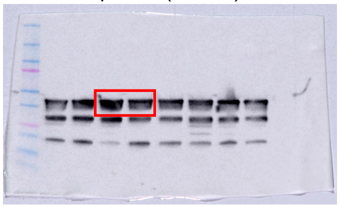

RATS WTvsCF

P-p65(65Dka)

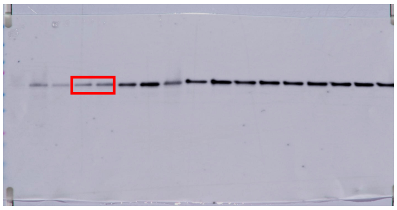

β-actin(44kDa)

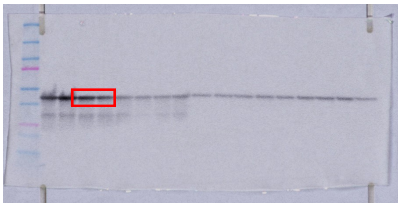

p65(65Dka)

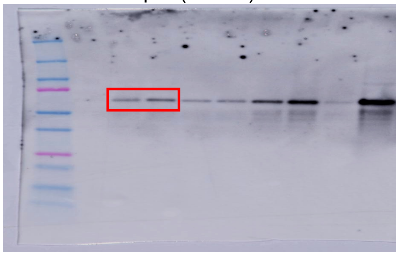

β-actin(44kDa)

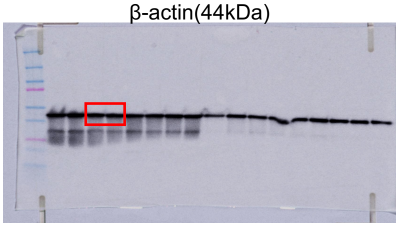

RATS CFvs CFAZD

P-p65(65Dka)

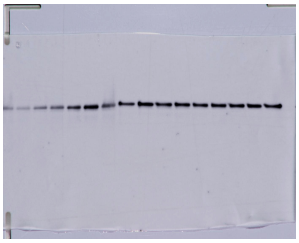

β-actin(44kDa)

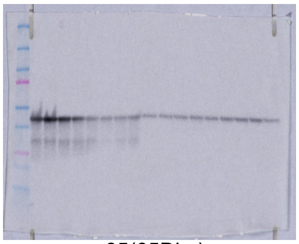

p65(65Dka)

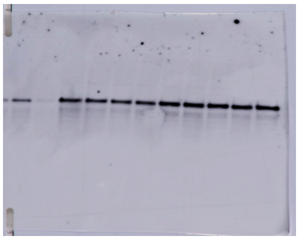

β-actin(44kDa)

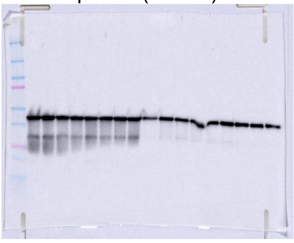

CAB

Blots for Supplemental Figure 12 A,B and C
